# Polyetheretherketone implants with hierarchical porous structure for boosted osseointegration

**DOI:** 10.1186/s40824-023-00407-5

**Published:** 2023-06-27

**Authors:** Zhiyong Chen, Yu Chen, Yang Wang, JiaJia Deng, Xin Wang, Qingqing Wang, Yuehua Liu, Jiandong Ding, Lin Yu

**Affiliations:** 1 State Key Laboratory of Molecular Engineering of Polymers, Department of Macromolecular Science, Shanghai Stomatological Hospital & School of Stomatology, Fudan University, Shanghai, 200438 China; 2 Department of Orthodontics, Shanghai Stomatological Hospital & School of Stomatology, Shanghai Key Laboratory of Craniomaxillofacial Development and Diseases, Fudan University, Shanghai, 200001 China; 3 Department of Orthopaedic Surgery, Sir Run Run Shaw Hospital, Medical College of Zhejiang University, Hangzhou, 310016 Zhejiang China

**Keywords:** PEEK, Surface modification, Biomaterials, Cold pressing, Hierarchical porous structure, Osseointegration

## Abstract

**Background:**

Good osseointegration is the key to the long-term stability of bone implants. Thermoplastic polyetheretherketone (PEEK) has been widely used in orthopedics; however, its inherent biological inertia causes fibrous tissue to wrap its surface, which leads to poor osseointegration and thus greatly limits its clinical applications.

**Methods:**

Herein, we developed a facile yet effective surface modification strategy. A commonly used sulfonation coupled with “cold pressing” treatment in the presence of porogenic agent formed a three-dimensional hierarchical porous structure on PEEK surface. Subsequently, the effects of porous surface on the in vitro adhesion, proliferation and differentiation of rat bone marrow-derived mesenchymal stem cells (BMSCs) were evaluated. Finally, the osteoinduction and osseointegration of surface-porous PEEK implant were examined in the rat distal femoral defect model.

**Results:**

In vitro results showed that the surface modification did not significantly affect the mechanical performance and cytocompatibility of PEEK substance, and the porous structure on the modified PEEK substrate provided space for cellular ingrowth and enhanced osteogenic differentiation and mineralization of BMSCs. In vivo tests demonstrated that the surface-porous PEEK implant could effectively promote new bone formation and had higher bone-implant contact rate, thereby achieving good bone integration with the surrounding host bone. In addition, this modification technique was also successfully demonstrated on a medical PEEK interbody fusion cage.

**Conclusion:**

The present study indicates that topological morphology plays a pivotal role in determining implant osseointegration and this facile and effective modification strategy developed by us is expected to achieve practical applications quickly.

**Graphical Abstract:**

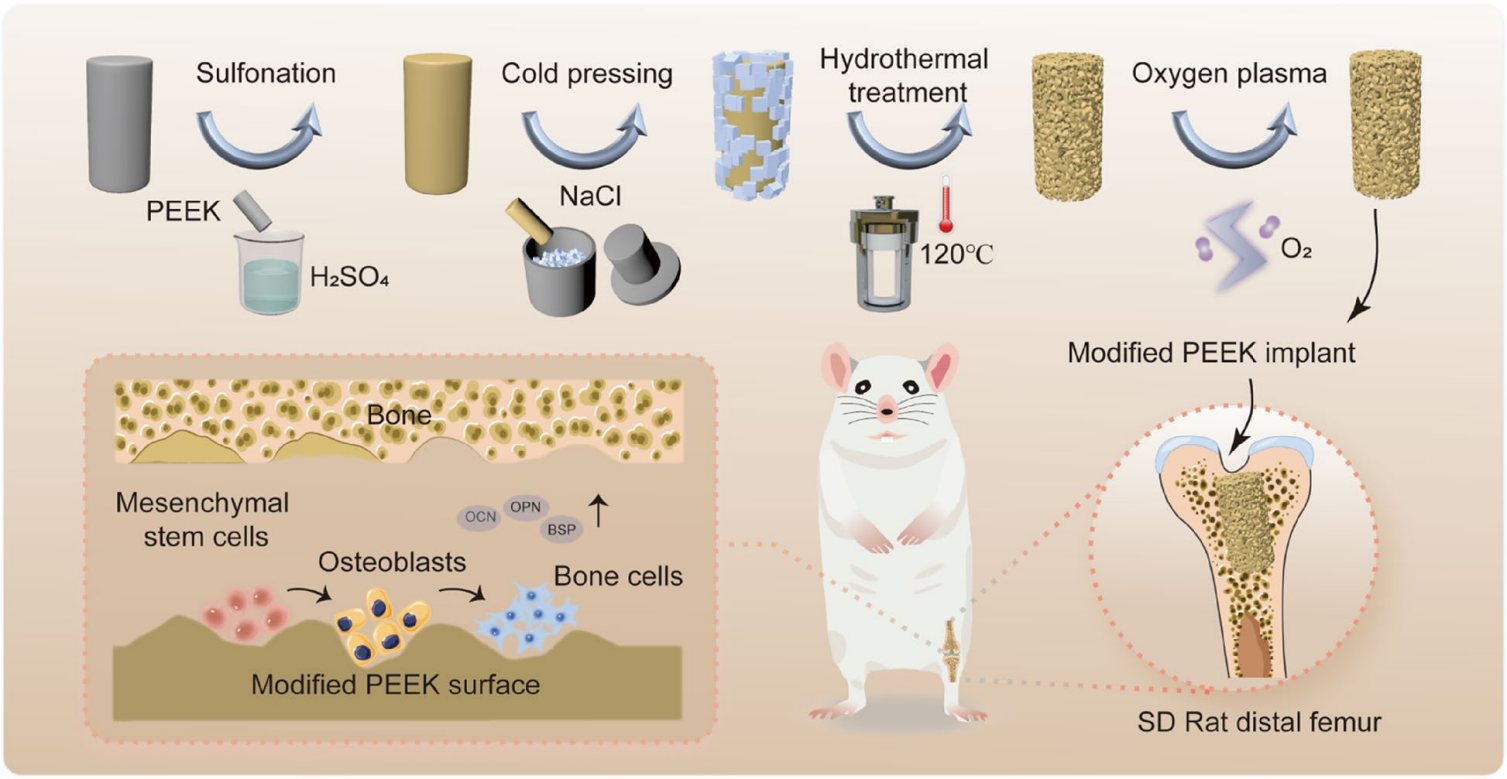

**Supplementary Information:**

The online version contains supplementary material available at 10.1186/s40824-023-00407-5.

## Introduction

Large segmental bone defect remains a vast clinical challenge, and due to its poor self-healing ability, orthopedic substitutes are often required [1, 2]. The raw materials used as bone substitutes mainly include metals, bioceramics, polymers and their composites [3–10]. Polyetheretherketone (PEEK) is a kind of synthetic semi-crystalline thermoplastic polymers. Since it was authorized by the U. S. Food and Drug Administration in 1998 as an orthopedic implant, PEEK has emerged as a preferred polymer material for spinal fusion, trauma management, joint and dental replacements, and craniomaxillofacial repair benefiting from its excellent biocompatibility, outstanding mechanical properties, high chemical stability as well as natural radiolucency [11–14]. Compared with conventional metal implants such as titanium alloys and stainless steels, PEEK has an elastic modulus closer to cortical bone, which effectively avoids the stress shielding effect and reduces the resorption of peripheral bone caused by modulus mismatch between the implant and the host bone [15–17]. However, the inherent biological inertia and poor bone induction of PEEK are the prime obstacles to its clinical application, which makes it difficult to induce new bone formation and achieve good osseointegration with the host bone, inevitably leading to the formation of fibrous cysts [11, 18, 19]. Eventually, PEEK implants become loose and fail, putting patients at risk for a secondary surgery.

To improve the osteogenic activity of PEEK, various modification methods have been developed, such as surface modification, blending of bioactive fillers, and so on [11, 14, 20–23]. Surface modification is an appealing strategy to boost the biological activity of PEEK without compromising its bulk mechanical properties [11, 21, 22]. Especially, incorporating bioactive coatings to PEEK surface has performed well both in vitro and in vivo [12, 24, 25], yet the poor stability of coatings and weak bonding to the substrate limited their application. A myriad of studies on titanium and other implantable materials have consistently validated that rough, porous surface topology is more conducive to osseointegration compared with smooth surface [26–29]. A few pertinent studies on PEEK surfaces support analogous findings [30–32]. In addition, surface porosity can accommodate bone ingrowth while averting tissue necrosis that is common in the center of bulk porous implants due to insufficient nutrient and vascular supplies [30, 33].

Sulfonation is an effective and commonly used approach to create a porous structure on PEEK surface, but only 0.5 to 10 μm micropores can be formed [34–36]. Some investigations have found that compared with smaller scale surface characteristics, larger scale characteristics may contribute more to the fixation of implants owing to enhanced mechanical interlocking [29, 32, 37, 38]. For example, PEEK with a 300–400 μm porous surface layer promoted cellular osteogenic differentiation and induced better osseointegration compared with smooth PEEK and PEEK modified with a plasma-sprayed titanium coating [32].

**Fig. 1:**
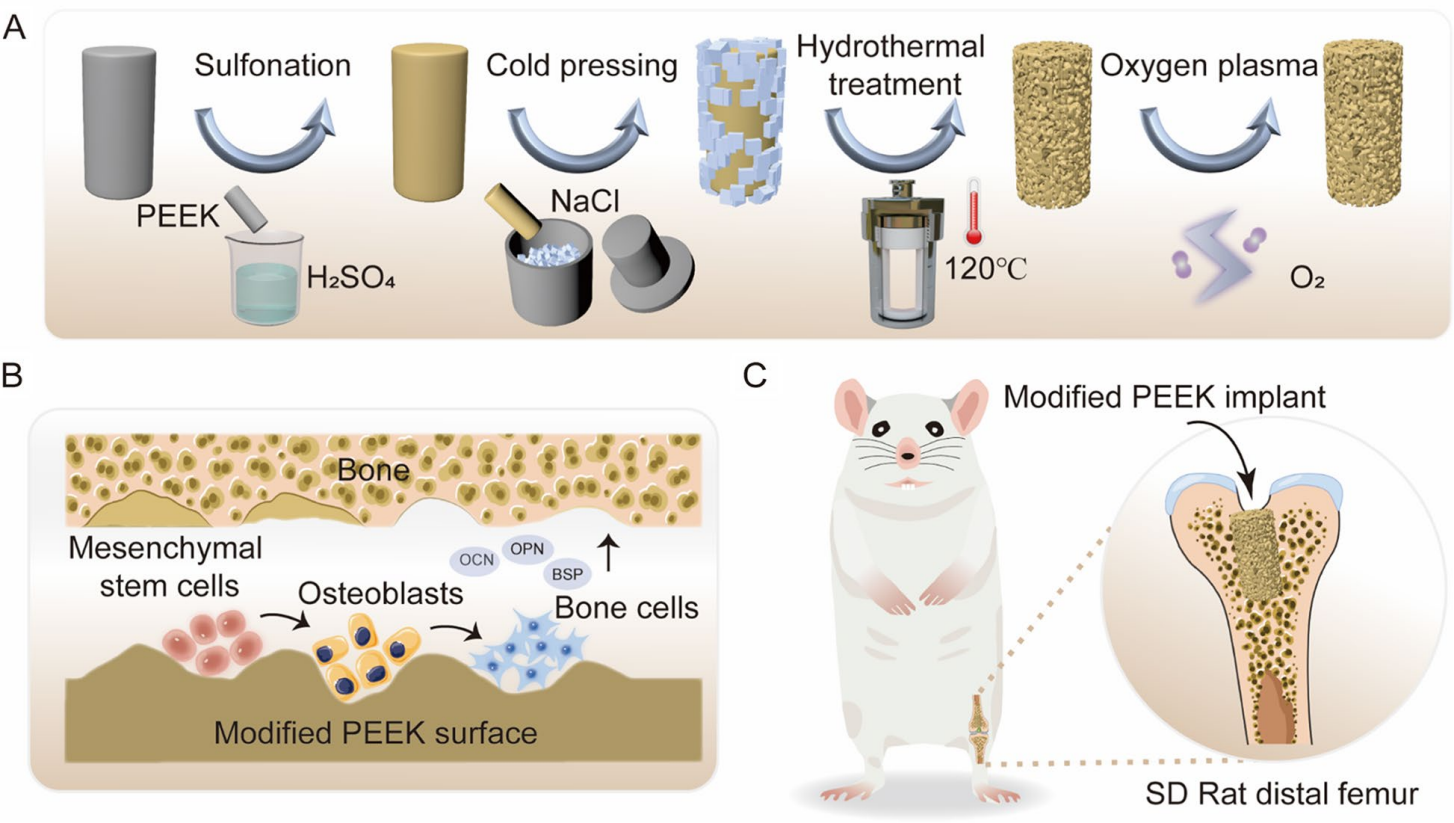
**A** Schematic illustration of preparation of PEEK surface with 3D hierarchical porous structure. **B** Enhancement of osteogenic differentiation of BMSCs on decorated PEEK surface. **C** Rat femoral defect model for evaluation of osteoinduction and osseointegration of decorated PEEK implant

Herein, we proposed a novel surface modification method to obtain a three-dimensional (3D) hierarchical porous architecture on PEEK surface, thus boosting its osteogenic activity and osseointegration. As illustrated in Fig. 1A, the sodium chloride (NaCl) porogenic agent was first embedded into the sulfonated PEEK surface through “cold pressing”. Subsequently, the porogenic agent and residual sulfuric acid were removed by hydrothermal treatment, so that the PEEK surface formed a hierarchical topological structure. In addition, the surface hydrophobicity could be improved by low-temperature oxygen plasma treatment. The morphology of PEEK surface before and after modification was characterized. The cytocompatibility of the modified PEEK was assessed. The proliferation and osteogenic differentiation of bone marrow-derived mesenchymal stem cells (BMSCs) on the modified PEEK substrate were detected (Fig. 1B). Finally, the in vivo bone induction and osseointegration capacity of the decorated PEEK implant was verified using a distal femoral defect model in Sprague Dawley (SD) rats (Fig. 1C).

## Materials and methods

### Preparation and surface modification of PEEK

PEEK samples of various sizes were prepared by hot pressing molding and the details are described in Fig. S1. First, PEEK powder (ZYPEEK, China) was added to the stainless steel mold and pressed at room temperature to form blanks. After that, the blanks were transferred into the preset molds (*Φ*10 × 1 mm, *Φ*15 × 1 mm, *Φ*34 × 1 mm, and *Φ*2 × 6 mm), whose upper and lower layers were covered with a polyimide membrane to prevent the sample contamination during processing. Next, the plate was clamped and placed in the plate vulcanizing machine. As the upper and lower template temperature rose to the preset temperature, the molding process was performed, including preheating, exhaust, molding, and cooling four steps. After the mold temperature dropped to room temperature, the polyimide membranes were carefully removed and then the PEEK samples were harvested by trimming and polishing.

The PEEK samples obtained were successively immersed in acetone, ethanol, and deionized water, and ultrasonic cleaning was performed for 10 min at each step. After drying, the disc-shaped (for in vitro tests) and cylindrical (for in vivo implantation) PEEK samples were incubated in concentrated sulfuric acid with a mass fraction of 95–98% for various times to soften the sample surface. Subsequently, the samples were subjected to “cold pressing” treatment in the presence of NaCl porogenic agent. Afterward, the samples were transferred into deionized water and hydrothermal treatment was conducted at 120 °C for 4 h to remove the residual sulfuric acid and porogenic agent. After drying at room temperature, some samples were further treated with low-temperature oxygen plasma (150 W, 15 min), followed by soaking in deionized water for 30 min and air-drying for subsequent use. The same modification process was employed for surface modification of medical PEEK interbody fusion cages.

### Surface topography observations

The surface morphologies of PEEK (unmodified), SP (sulfonation and hydrothermal treatment), SPC (cold pressing and hydrothermal treatment of SP) and SPCP (low-temperature oxygen plasma treatment of SPC) were visualized by a field emission scanning electron microscope (FE-SEM, Zeiss). The 3D topography observation and two-dimensional (2D) linear height scanning of the sample surface were carried out via a step profilometer (AlphaStep D-600, KLA-Tencor Corp.).

### Analysis of surface properties

The surface groups of various PEEK samples were analyzed via Fourier transform infrared spectroscopy (FTIR, Nicolet 6700, Thermofisher) and X-ray energy dispersive spectroscopy (SEM–EDS). The compressive properties of PEEK platelets (10 × 10 × 4 mm) were tested using a universal testing machine with 1 mm/min test speed (Instron 5966). The PEEK surface roughness was assayed by a step profilometer. To determine the wettability of PEEK surface, the static contact angles of water and diiodomethane on various sample surfaces were measured using an optical contact angle measuring device (JC2000DM, Zhongchen). Subsequently, the total surface energy was calculated using the Owens two-liquid method [39]. The shore hardness of various samples was detected by a D-type shore hardness tester (LX-D, Wenzhou Weidu Electronic Co., Ltd.).

Fibronectin (FN) was used to analyze the protein adsorption capacity of PEEK surfaces. The specimens were immersed in 25 μg/mL FN solution. After incubation at 37 °C for 4 h, the specimens were gently washed twice with phosphate buffered saline (PBS) to remove the unadsorbed proteins, and then incubated in 2% sodium dodecyl sulfate (SDS) solution at 37 °C for 1 h to desorb the absorbed proteins. Finally, the protein concentration in the SDS supernatant was quantified by the BCA protein assay kit (Beyotime, China).

### In vitro cell experiments

BMSCs were separated from SD rats (3–4 weeks old) and the details are illustrated in Fig. S2. For cell culture, 10% fetal bovine serum (FBS, Gibco, USA), 100 U/mL of penicillin (Gibco), 100 μg/mL of streptomycin (Gibco) and 100 μg/mL minimum essential medium α (α-MEM, Gibco) were added. All cells were cultured in an incubator (37 °C, 5% CO_2_) and passaged at 80–90% confluency. Culture medium was replaced every 2–3 days. All samples used for cell experiments were sterilized with 75% ethanol plus UV irradiation.

BMSCs (2 × 10^4^/well) were seeded on the PEEK samples (*Φ*10 × 1 mm) in 48-well plates and cultured for 12 h. After removing the medium, the samples were washed three times with PBS. Then, the cells on samples were fixed with 4% paraformaldehyde solution at room temperature for 15 min, followed by cleaning with PBS to remove residual paraformaldehyde solution. Next, the samples were dehydrated with gradient ethanol solution (10%, 20%, 30%, 50%, 70%, 80%, 90%, 100%) for 15 min. Finally, SEM was used to observe the adhesion and spreading of cells on the PEEK surface. In addition, the nuclei and cytoskeleton of fixed cells were stained with 2-(4-amidinophenyl)-6-indolecarbamidine dihydrochloride (DAPI, Beyotime, China) and rhodamine-phalloidin (Beyotime), respectively. The adhesion morphology of BMSCs on the PEEK surface was then observed by a confocal laser scanning microscope (CLSM, Nikon).

Cytocompatibility of PEEK surface was assessed by a live & dead viability/cytotoxicity assay kit consisting of calcein-AM and propidium iodide (PI, Beyotime, China). BMSCs (2 × 10^4^/well) were seeded on the PEEK samples in 48-well plates. After 1 or 3 days of culture, PBS containing live/dead assay reagent was added and incubated in the dark for 30 min. After carefully washing 3 times with PBS, CLSM was used to observe the cells on the samples.

BMSCs (1 × 10^4^/well) were cultured on the PEEK surface in 48-well plates, and the medium was replaced every two days. After 1, 4 and 7 days of culture, the cell counting kit-8 (CCK-8) assay was used to detect cell viability and proliferation [40, 41]. At each checkpoint, the medium was replaced with fresh medium incorporating 10% CCK-8 assay reagent (Beyotime, China) and incubated at 37 °C for 2.5 h. 100 μL of the supernatant was transferred into a 96-well plate and the absorbance at 450 nm was measured by a microplate reader (ELx808, BioTek). In addition, at each time point, the nuclei and cytoskeleton of fixed cells were stained with DAPI and rhodamine-phalloidin, respectively. Subsequently, the BMSCs on the PEEK surface were observed using CLSM.

### In vitro evaluation of osteogenic differentiation

BMSCs (5 × 10^4^/well) were seeded on various PEEK samples (*Φ*15 × 1 mm) in 24-well plates. After the cells grew to 90% confluence, the medium was replaced with osteogenic induction medium every two days. The osteogenic induction medium was obtained by adding 10% FBS, 50 μM ascorbic acid, 10 mM β-glycerophosphate and 100 nM dexamethasone to α-MEM.

After 3 and 7 days of osteogenic induction, the media were removed, and the samples were then gently rinsed 3 times with PBS. After that, the cells were fixed with 4% paraformaldehyde at room temperature for 15 min, and the residual paraformaldehyde was removed by washing with PBS for 3 times. Then, a BCIP/NBT alkaline phosphatase (ALP) color development kit (Beyotime, China) was added to stain the samples, as stated in the manufacturer’s instructions. Finally, the stained samples were scanned using a Laser Jet Pro MFP M227fdw (HP, China). In addition, the ALP activity of BMSCs on different samples was quantitatively detected by an ALP assay kit (Beyotime, China). In detail, the cells were lysed with cell lysis solution, followed by centrifugation to collect the supernatant. Para-nitrophenyl phosphate (pNPP), an ALP chromogenic substrate, was added to the supernatant and incubated at 37 °C and in the dark for 10–30 min. Next, the absorbance at 405 nm was detected by a microplate reader under alkaline conditions. At the same time, the total protein concentration of each group was determined by a BCA protein assay kit (Beyotime, China). ALP activity was obtained after normalization to total protein content.

After 14 and 21 days of osteogenic differentiation, mineralized nodule formation was confirmed using an Alizarin red-S (ARS) staining kit (Beyotime, China). Briefly, the medium was discarded, and the cells were gently washed 3 times with PBS. Subsequently, the samples were fixed at room temperature for 15 min and stained with ARS solution for 30 min. After that, the excessive stain was rinsed off with deionized water. The photographs of stained samples were captured by a Laser Jet Pro MFP M227fdw. For quantitative evaluation of the degree of mineralization, 10% cetylpyridinium chloride (Adamas, China) was used to dissolve the red mineralized nodules and the absorbance at 562 nm was detected by a microplate reader.

The expression levels of osteogenic property-related genes were analyzed by quantitative reverse transcription polymerase chain reaction (qRT-PCR). First, BMSCs (2 × 10^5^/well) were seeded on various samples (*Φ*34 × 1 mm) in 6-well plates. After 7 days of osteogenic induction, total RNA was extracted by a FastPure cell/tissue total RNA isolation kit (Vazyme, China) and RNA concentration was determined by Nanodrop. After reverse transcription using a Prime Script RT reagent kit (Takara, Japan) to synthesize cDNA, qRT-PCR analysis was performed using a QuantiNova SYBR Green PCR Kit (Qiagen, USA). The sequences of the primers for osteopontin (OPN), osteocalcin (OCN) and bone sialoprotein (BSP) are listed in Table S1. The 2^−ΔΔCt^ method was used to calculate the relative expression levels of genes. The fold change of the relative gene expression was normalized to the PEEK group.

### Surgical procedure

Female SD rats (8 weeks old) were bought from Shanghai Lab. Animal Research Center. The animals received 12 h of light and dark cycle daily and were supplied with plenty of food and water. The animal experiment was approved by the Ethics Committee of the Laboratory Animal Science Department of Fudan University (202201015S). A total of 24 SD rats were randomly divided into 4 groups (PEEK, SP, SPC, and SPCP). All implants were sterilized with 75% ethanol plus UV irradiation before use. General anesthesia was performed on SD rats by intraperitoneal injection of 2% pentobarbital assisted by gas anesthesia machine. A sagittal incision was made on the skin of the distal femur, and the femoral condyle was separated and exposed. A cylindrical defect of *Φ*2 × 6 mm was created along the femoral shaft using a dental drill, and the wound was then carefully sutured after filling an implant. For the initial 5 days after surgery, intraperitoneal injection of 2 × 10^5^ U/kg penicillin G sodium solution was performed daily. ARS (30 mg/kg), tetracycline hydrochloride (25 mg/kg) and calcein (20 mg/kg) were injected intraperitoneally at 2, 4 and 6 weeks post-operation, respectively. After 8 weeks of operation, the rats were euthanized. The harvested femur samples were fixed in 4% paraformaldehyde solution for subsequent evaluation.

### μ-CT analysis

The femurs containing implants were scanned via the μ-CT Skyscan 1276 system (Bruker, Germany). Scan settings are as follows: voxel size 9.0 μm, energy setting 85 kV, 200 μA, 1 mm Al filter and integration time 384 ms. Reconstruction was carried out by NRecon software (version 1.7.4.2). 3D images were obtained from contoured 2D images by methods based on distance transformation of the grayscale original images (CTvox; version 3.3.0). CT Analyser (version 1.18.8.0) was used to analyze the region of interest (ROI), including the 200 μm annular region extending around the implant. The bone-implant contact (BIC), total volume (TV), bone volume (BV), volume ratio (BV/TV), bone surface (BS), bone mineral density (BMD), trabecular bone number (Tb.N), trabecular separation (Tb.Sp), and trabecular thickness (Tb.Th) were calculated by software.

### Histological analysis

After μ-CT analysis, some femur specimens were dehydrated in a gradient ethanol series (70–100%), and then embedded in methyl methacrylate solution at 37 °C. Next, a microtome (EXAKT310, Germany) is used to prepare the femoral sections with 200 μm thickness along the direction of the implant. Micrographs of fluorescent markers around the implant were captured by CLSM. Finally, the samples were stained with methylene blue-fuchsin to observe bone ingrowth and integration around the implants. On the other hand, other femur samples were decalcified in 10% ethylenediaminetetraacetic acid (EDTA) for 30 days. The decalcified samples were dehydrated by gradient ethanol, embedded in paraffin and sectioned into 5 μm thickness. Afterwards, some sections were subjected to Hematoxylin–Eosin (H&E) and Masson’s trichrome staining. Other sections were further deparaffinized and then stained with Goldner’s trichrome. All stained sections were imaged by an inverted optical microscope (DS-U3, Nikon). Additionally, to detect the activity of bone formation, the expression of osteogenic-related proteins RUNX2 and OCN was analyzed via immunofluorescence staining according to previously described procedures [42]. The samples obtained were visualized via an inverted fluorescence microscope (E100, Nikon).

### Statistical analysis

All data are expressed as mean ± standard error of mean. Statistical analysis between different groups was performed using one-way analysis of variance (ANOVA) followed by Tukey’s post hoc test for multiple comparisons. A statistically significant difference was accepted at *p* < 0.05 as usual.

## Results

### Construction and characterization of hierarchical porous structure on PEEK surface

PEEK surface with 3D hierarchical topological structure was facilely obtained by sulfonation coupled with “cold pressing” treatment. Sulfonation is a commonly used method for the surface modification of PEEK-based implants [34–36]. Interestingly, we found that sulfonation treatment could soften PEEK surface temporarily. Inspired by this, the “cold pressing” strategy was suggested for the first time to embed porogenic agent into PEEK surface, as shown in Fig. 1A. First, crushed NaCl particles were sieved to obtain porogenic agent of expected size. Then, the PEEK sample sulfonated with concentrated sulfuric acid was transferred into a mold containing the porogenic agent, which was pressed into the PEEK surface at room temperature. The influence of sulfonation time on the embedding effect of porogenic agent on PEEK surface was also assessed. When the sulfonation time was less than 2 min, the porogenic agent could not be effectively embedded into the PEEK surface because its surface was not completely softened, as depicted in Fig. S3. As the sulfonation time was extended to 2 min and longer, the porogenic agent was easy to embed into the PEEK surface. Considering the cost performance, the sulfonation time was fixed at 2 min in the subsequent study.

**Fig. 2:**
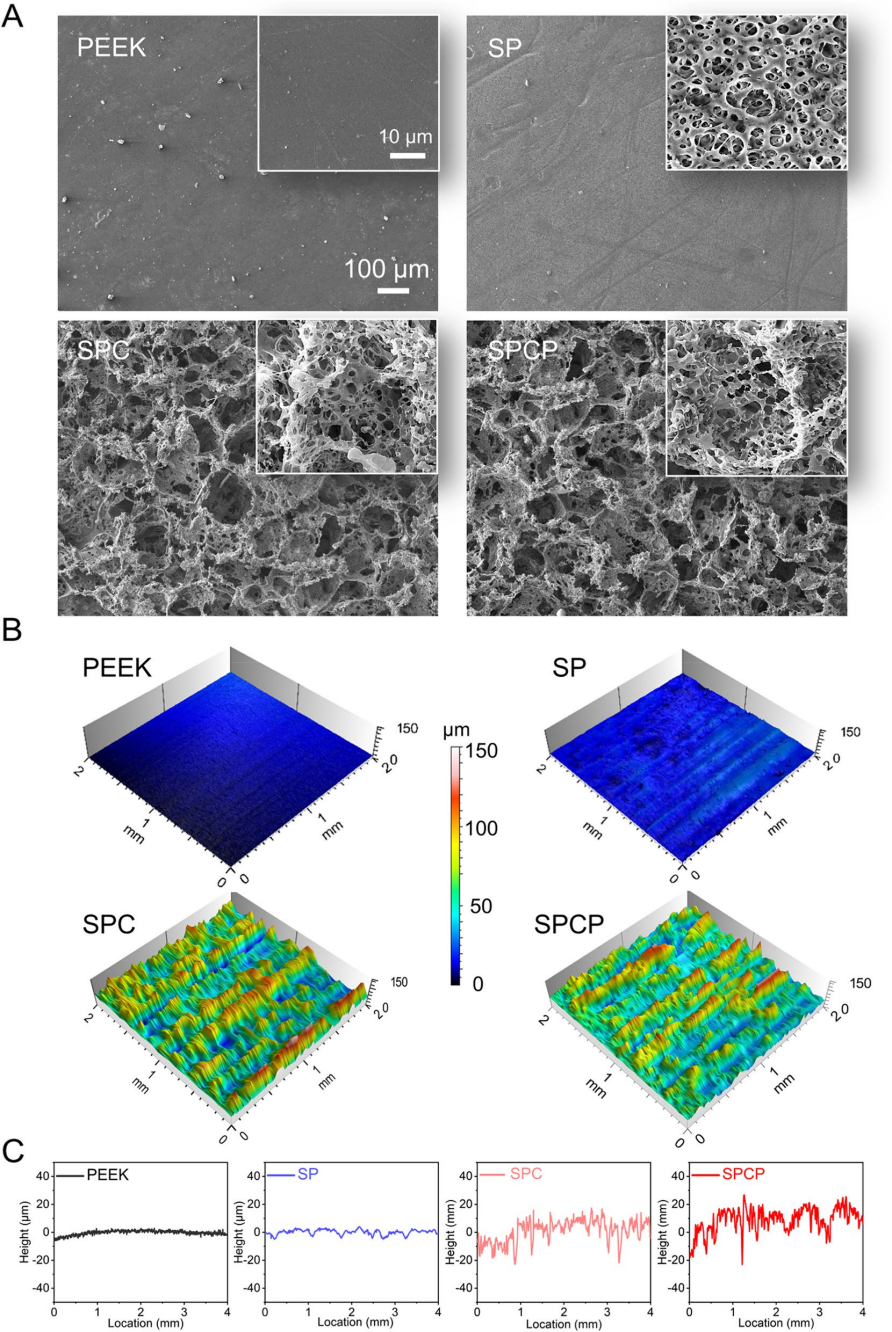
Surface topography characterization. **A** SEM images of various samples. **B** 3D reconstructed surface morphology. **C** Height scanning of various samples

SEM is a potent tool to investigate the surface morphology of implants [43, 44]. Figure 2A shows the SEM images of surface morphology of PEEK before and after modification. At low magnification, the untreated and sulfonated PEEK surfaces were relatively smooth. With increasing magnification, 0.5–10 μm multi-layered pore structure was seen on the sulfonated PEEK surface, as previously reported [31, 34, 45]. The surfaces of SPC and SPCP showed 100–200 μm pore structure, which was consistent with the size of NaCl particles, and the sulfonation-induced 0.5–10 μm small pores were also well retained. Meanwhile, there was no significant difference between the two substrate surfaces, indicating that the low-temperature oxygen plasma treatment did not compromise the hierarchical porous structure on their surfaces.

The topographical features of various sample surfaces were further analyzed via a step profilometer. As presented in Fig. 2B, compared with PEEK and SP with smooth surface, the surfaces of SPC and SPCP treated with “cold pressing” became rugged, which was in line with the SEM images. The height scanning of sample surface also confirmed that small craters appeared on the SP surface, while large deep pits were observed on the SPC and SPCP substrates (Fig. 2C). Further observation of the cross-sectional morphology showed that there was a sulfonation layer with a thickness of about 30 μm on the SP surface (Fig. S4A). Meanwhile, both the height scanning and cross-sectional SEM images proved that the depth range of large pore was about 20–50 μm (Figs. 2C and S4A).

**Fig. 3:**
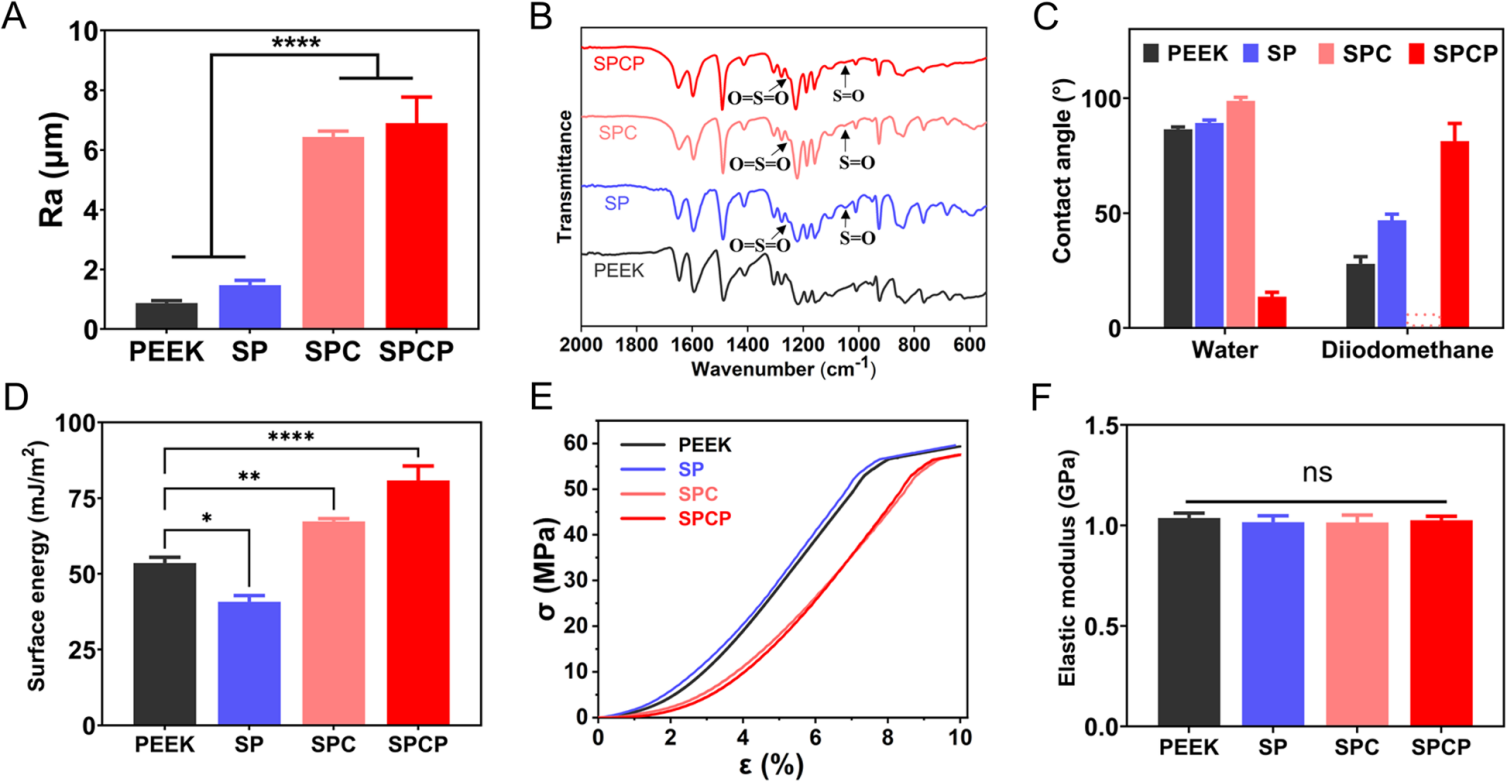
Characterization of surface properties. **A** Surface roughness of different samples (*n* = 4). **B** FTIR spectra of various sample surfaces. **C** Contact angles of water and diiodomethane on different sample surfaces (*n* = 6). **D** Total surface energy calculated by the Owens two-liquid method using the data of contact angle (*n* = 6). **E** Compressive stress–strain curves of different samples. **F** Compression moduli of different samples (*n* = 3)

Quantitative micro-topographical analysis showed that the surface roughness of SPC and SPCP was substantially higher than that of PEEK and SP (Fig. 3A), which further supported the above results. In addition, the 3D hierarchical porous structure on PEEK surface could be easily adjusted by changing the size of NaCl particles, indicated that the modification method developed by us has good controllability (Fig. S4B).

Figure 3B displays the FTIR spectra acquired from the indicated samples. The appearance of the O = S = O asymmetric stretching vibration peak at 1255 cm^−1^ and the S = O symmetric stretching vibration peak at 1050 cm^−1^ validated the presence of sulfonic acid groups (-SO_3_H) on the surfaces of SP, SPC and SPCP, which was consistent with previous studies [31, 46]. Meanwhile, EDS analysis manifested that although the S content on the substrate surface pronouncedly increased after sulfonation and then significantly decreased with hydrothermal treatment, the S amount on the SP surface after hydrothermal treatment was still substantially higher than that of pristine PEEK (Fig. S5). This result indicated that the residual H_2_SO_4_ could be removed via hydrothermal treatment, but the covalently bonded –SO_3_H groups on the PEEK surface were well retained.

The contact angles of water and diiodomethane on PEEK surface before and after various treatments are presented in Fig. 3C. The water contact angles of PEEK, SP, SPC, SPCP were 86.5°, 89.2°, 98.8°and 13.6°, respectively, while their contact angles of diiodomethane were 27.9°, 46.9°, 0° and 81.3°, respectively. Although the surface hydrophobicity of PEEK gradually increased after sulfonation and/or “cold pressing” treatment, further plasma treatment could dramatically boost the surface hydrophilicity of SPCP (Figs. 3C and S6). In general, hydrophilic surface of implants facilitates initial blood contact, promotes cell adhesion, stimulates cell proliferation and differentiation, thus accelerating bone formation and osseointegration [19, 47]. In addition, the implant’s surface energy can reflect its surface activity to a certain extent [48, 49]. Therefore, the total surface energy of each sample was calculated using the contact angles of water and diiodomethane. As displayed in Fig. 3D, the SPCP sample had the highest surface energy, followed by SPC, PEEK and SP.

The effect of surface modification on the mechanical properties of PEEK implants was also appraised. While the “cold pressing” treatment shifted the stress–strain curves of SPC and SPCP slightly to the right (Fig. 3E), their compressive moduli had no notable difference, similar to that of pristine PEEK (Fig. 3F). Meanwhile, given that matrix hardness is one of the pivotal biophysical signals to regulate stem cell differentiation [50], the shore hardness of each sample was also measured. As presented in Fig. S7, the shore hardness values of PEEK, SP, SPC, SPCP were 81.3, 79.4, 78.0 and 77.9 HD, respectively, indicating that various surface modifications slightly decreased the shore hardness of PEEK substance. Overall, this modification strategy developed by us had no significant effect on the mechanical properties of PEEK.

### Observations of cells on modified PEEK surfaces

**Fig. 4:**
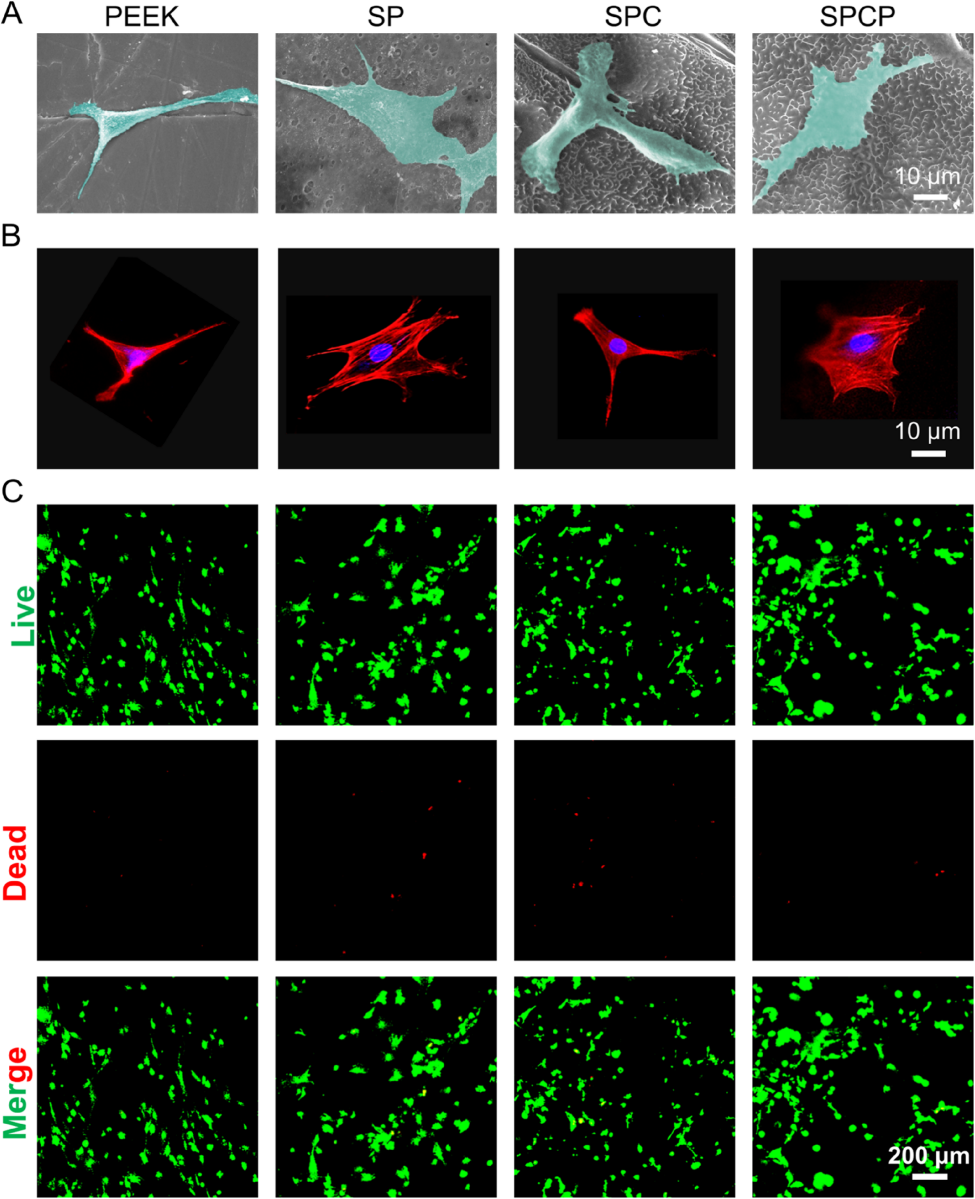
In vitro cell adhesion and cytocompatibility. **A** SEM images of BMSCs on different substrates after culturing for 12 h. **B** The corresponding CLSM images. **C** Fluorescence images of calcein-AM/PI double stained BMSCs on diverse samples after culturing for 1 day. Calcein-positive cells: green; PI-positive cells: red

BMSCs are considered as the ideal cells for assessing osteogenic performance of orthopedic substitutes, given their pivotal contributions to bone integration [34, 51, 52]. First, the adhesion of BMSCs on different samples was observed by SEM and CLSM. After 12 h of culture, BMSCs only exhibited triangular or spindle morphology on the PEEK and SPC substrates (Fig. 4A-B), suggesting that the cells were reluctant to adhere to their surfaces. In contrast, BMSCs in the SP and SPCP groups were polygonal in shape and had more pseudopodia and larger spreading areas in contact with the substrate surface. Especially, the cells adhered firmly to the SPCP surface and generously spread with many filopodia anchored into the pores, which might result from the 3D hierarchical porous structure plus increased hydrophilicity.

The cytotoxicity of various samples against BMSCs was also measured by the live & dead viability/cytotoxicity assay. As displayed in Fig. 4C, most BMSCs were alive, although a few scattered dead cells appeared on the surfaces of SP, SPC and SPCP. This feature suggested that surface modification did not prominently affect the cytocompatibility of PEEK matrix.

**Fig. 5:**
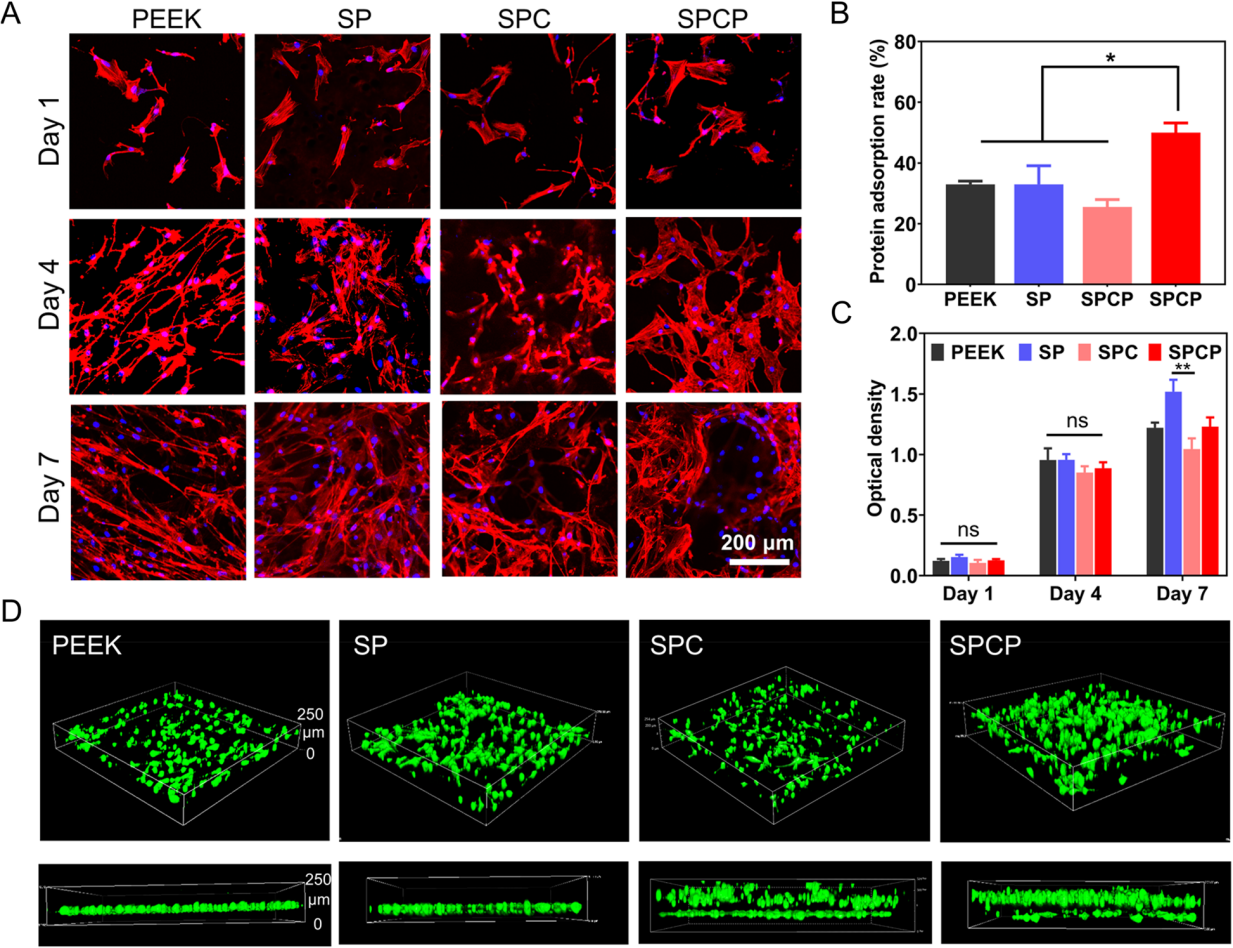
In vitro cell growth and proliferation. **A** CLSM images of BMSCs seeded on the indicated samples at different time points. **B** Protein adsorption rate in each group (*n* = 4). **C** CCK-8 results of BMSCs cultured on the indicated samples at different time points (*n* = 4). **D** 3D and Z-directional CLSM images of BMSCs on various samples after 3 days of culture

The time-dependent proliferation of BMSCs on diverse substrates was detected via CLSM and the CCK-8 assay. As shown in Fig. 5A, BMSCs proliferated rapidly and had completely covered all the substrate surfaces with the incubation time extended to 7 days. CLSM observations further confirmed that BMSCs had better early adhesion and spreading ability on the SPCP substrate than the other groups.

Compared with the others, the SPCP substrate induced more protein adsorption (Fig. 5B) due to its excellent hydrophilicity and high specific surface area, which was also one of the contributing factors that promoted cell adhesion and spreading. Figure 5C shows the quantitative results of CCK-8 assay. At each time point, the OD values of the modified samples had no notable difference compared with that of the pristine PEEK, which coincided with the results of CLSM observation.

In addition, both the 3D reconstructed CLSM images and the Z-axis stacked images distinctly manifested that unlike cells growing on the PEEK and SP surfaces, BMSCs grew into the 3D hierarchical porous structure of SPC and SPCP substrates (Figs. 5D and S8). This feature provided favorable conditions for early osteogenic fixation.

### Promoted osteogenic differentiation of BMSCs in vitro

In general, cell differentiation is regarded to be a critical step in damage repair, and then differentiated cells will undergo a battery of conversions and ultimately form functional tissue. BMSCs are a population of pluripotent stem cells with the ability of multidirectional differentiation [42, 51]. Consequently, we also investigated the effect of 3D hierarchical topological structure on osteogenic differentiation of BMSCs.

**Fig. 6:**
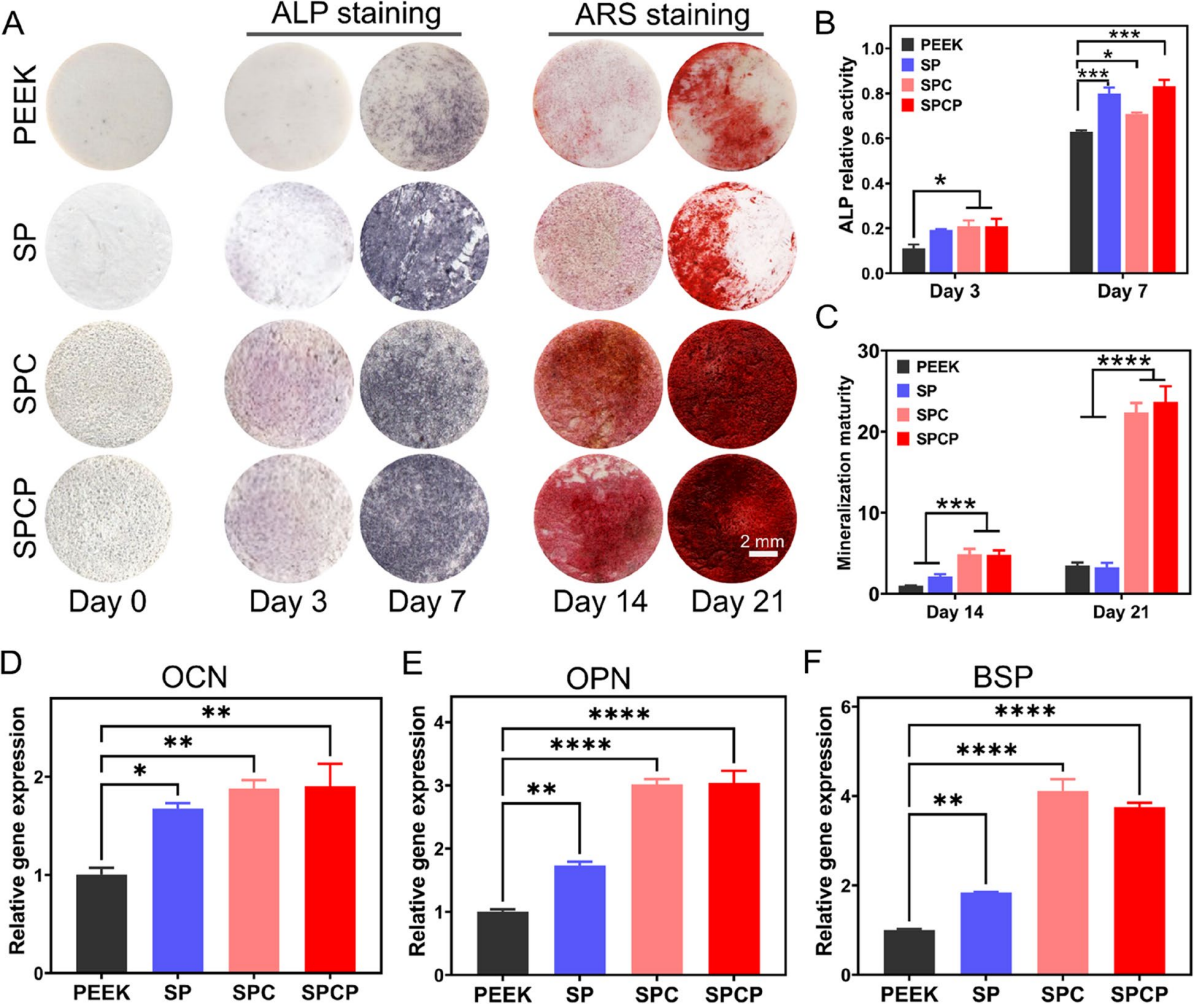
In vitro differentiation and mineralization of BMSCs induced by different substrates. **A** Representative photographs of ALP and ARS staining of BMSCs cultured on different groups at the indicated time points. **B** ALP relative activity of BMSCs on different surfaces at 3 and 7 days after culturing (*n* = 4). **C** Quantification of mineralized nodules at 14 and 21 days after culturing (*n* = 4). Relative expression of osteogenesis-related genes **D**) OCN, **E**) OPN, and **F**) BSP at 7 days after culturing (*n* = 3)

ALP activity is considered to be an important indicator of early osteogenic differentiation, which is involved in the calcification process of new bone and affects the mineralization ability of osteogenic differentiated cells [42, 45, 53, 54]. Therefore, the ALP expression of BMSCs cultured on different substrates was detected via ALP staining to determine whether the substrates had the ability to expedite the formation of new bone. As shown in Fig. 6A, ALP activity gradually increased in each group with the passage of incubation time. Moreover, ALP expression was prominently higher in the SP, SPC and SPCP groups compared with the untreated PEEK group, which was further validated via the quantitative results (Fig. 6B). These findings suggested that these surface modifications were beneficial for early osteogenesis.

The formation of mineralized nodules is a sign of osteoblast differentiation and maturation [42, 45]. At 14 and 21 days after culturing, ARS staining and quantitative analysis were conducted to confirm the mineralized potency of diverse samples. PEEK and SP only showed limited mineralization efficiency after 14 and 21 days of osteogenic induction (Fig. 6A). Conversely, the SPC and SPCP substrates induced a dramatic increase in the deposited area of calcium nodules. The quantitative outcomes also well supported this finding (Fig. 6C), indicating that the 3D hierarchical porous structure facilitated calcium deposition and extracellular matrix mineralization.

In addition, qRT-PCR results showed that compared with the PEEK group, the expression of OCN, OPN and BSP genes in BMSCs, which are closely related to osteogenesis, was significantly up-regulated in the other groups after 7 days of culture, especially in the SPC and SPCP groups (Fig. 6D-F).

### Accelerated bone formation and osseointegration in vivo

**Fig. 7:**
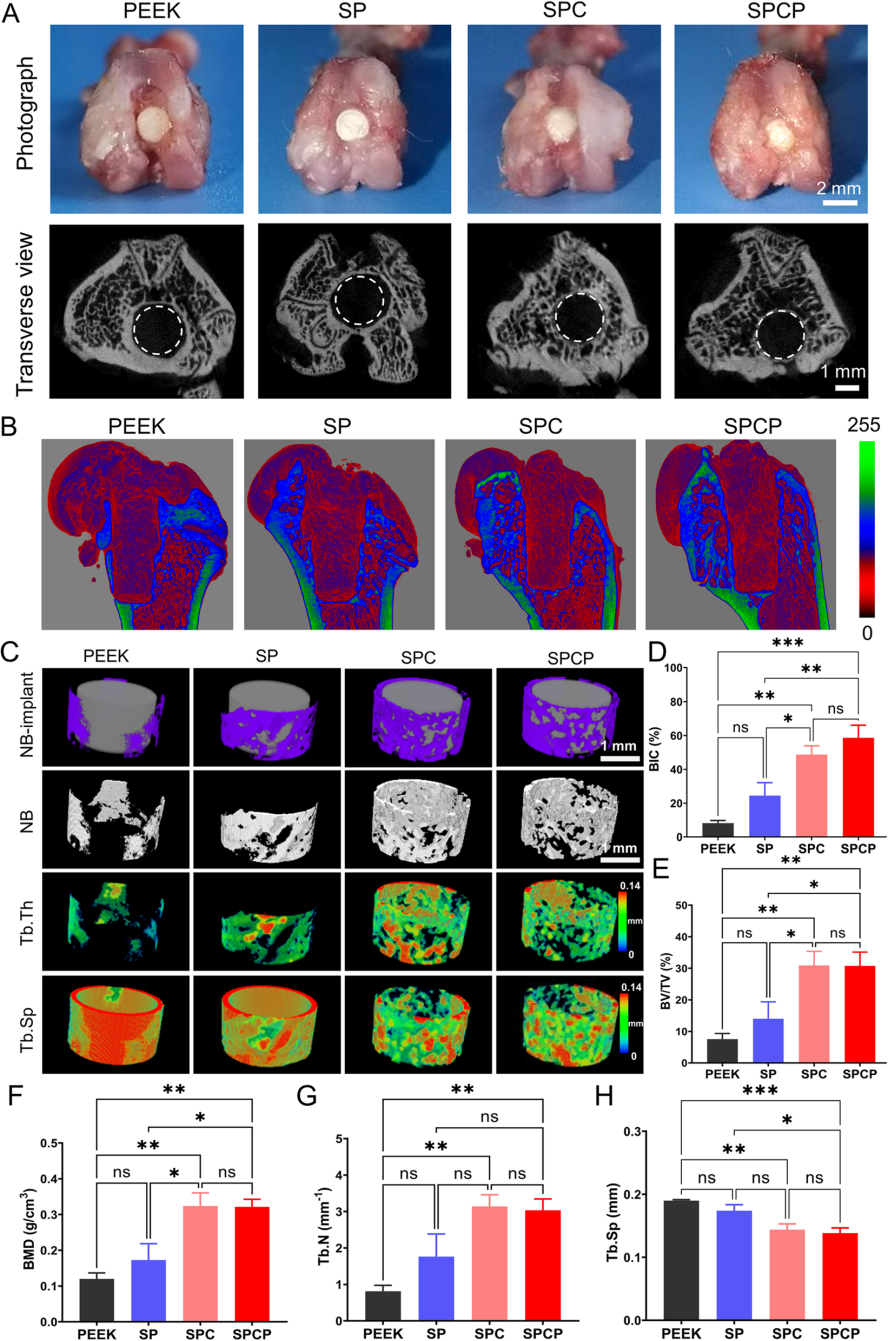
In vivo bone repair results in the rat distal femoral defect model. **A** Representative macroscopic photographs of the femurs containing PEEK implants at 8 weeks post-implantation and corresponding 2D μ-CT images from transverse view. **B** 3D reconstructed μ-CT images of the femurs containing implants at 8 weeks post-implantation. The scalebar represents the gray value. The greater the gray value, the higher the bone density. **C** 3D reconstructed μ-CT images of the adjacent bone tissues with or without implants at 8 weeks post-implantation. Quantitative analysis of μ-CT data including **D**) BIC (*n* = 5), **E**) BV/TV (*n* = 6), **F**) BMD (*n* = 6), **G**) Tb.N (*n* = 6), and **H**) Tb.Sp (*n* = 6)

Encouraged via the results of in vitro experiments, we created a distal femoral defect model in SD rats to corroborate the osteoinduction and osseointegration of hierarchical porous PEEK implants. After 8 weeks of implantation, the animals were humanely sacrificed and the femurs containing implants were gathered for histomorphometric evaluation. From the perspective of optical photographs (Fig. 7A), all implants were embraced by surrounding tissues and no obvious complications such as infection and displacement were observed. However, from the transverse views of 2D μ-CT images, there was a distinct gap between the adjacent bone and the implant in the PEEK and SP groups, indicating the poor osseointegration (Fig. 7A).

CT has been extensively used to observe the regeneration of bone tissue around implants and to estimate the osseointegration of implants [55, 56]. Therefore, the regenerated bone tissues surrounding various PEEK implants were reconstructed using μ-CT. Different from metals, the inherent radiolucency of PEEK substance allows for a more intuitive assessment of new bone formation around PEEK-based implants. Figure 7B shows the μ-CT reconstructed femur tissues containing implants. During bone regeneration, osteoblasts differentiated from BMSCs first secrete unmineralized collagen (osteoid), and then mature into osteocytes surrounded via mineralized bone matrix, so new bone density is generally less than mineralized bone density [15, 57, 58]. That is, newborn bone has a smaller gray value than mineralized bone. It can be seen that the red areas around the implants in the SPC and SPCP groups are larger than in the other two groups, which seems to hint that more new bone was formed. To get a better look at the formation of new bone in the peri-implant region, the μ-CT reconstructed ring structure extending 200 μm from the implant periphery was also acquired. As displayed in Fig. 7C, there was only some new bone surrounding the PEEK and SP implants. Conversely, both the SPC and SPCP implants were enwrapped with a large amount of newly formed bone at 2 months post-operation, indicating that they did have stronger osteogenic ability in vivo.

Subsequently, the parameters reflecting the quality of new bone formation, including BIC, BV/TV, BMD, BS/TV, Tb.N, Tb.Sp, and Tb.Th, were quantified using the 3D reconstructed μ-CT images. The BIC, BV/TV, BMD and BS/TV in the SPC and SPCP implants were prominently higher compared with the other two groups (Figs. 7D-F and S9A), and there was no noteworthy difference between the SPC group and the SPCP group. Thereinto, the BV/TV in the SPC and SPCP groups was 4.1 and 2.2-fold remarkably higher than that of the PEEK and SP groups, respectively. In addition, an increase in the Tb.N and a reduction in the Tb.Sp, reflecting the distance between adjacent trabecula, were also observed in both the SPC and SPCP groups, though there was no considerable difference in the Tb.Th among all groups (Figs. 7G-H and S9B). These favorable results confirmed that the newly formed bone around the SPC and SPCP implants were evidently superior to the other two groups in morphology and morphometry.

**Fig. 8:**
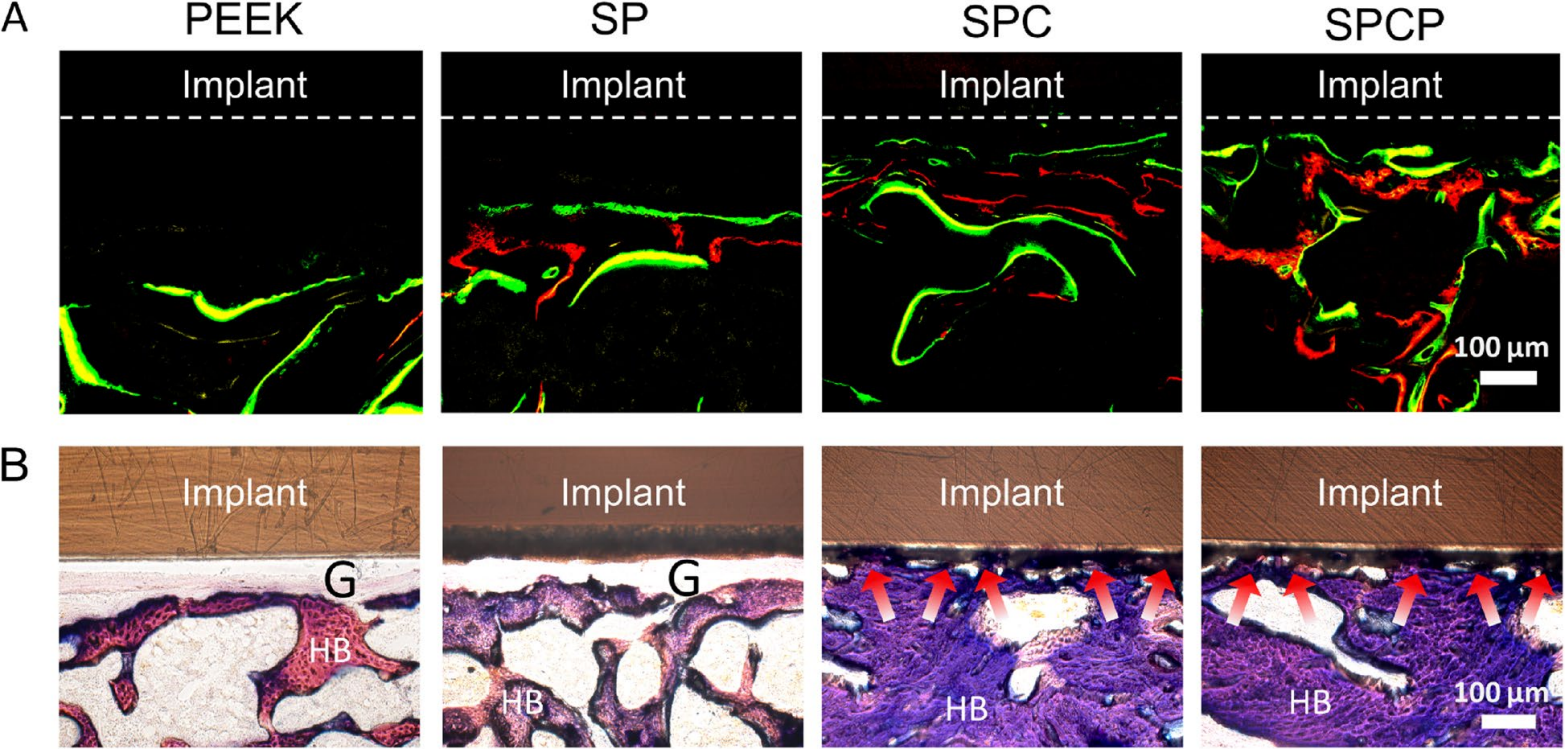
Histological evaluation of osseointegration after various treatments. **A** CLSM images of sequential fluorescence labeled bone tissue around the implants. The dotted lines show the boundary between the implant and the surrounding tissue. **B** Methylene blue-acid fuchsin staining sections. The red arrows indicate newborn bone ingrowth into the implants. G: gap; HB: host bone

To further assess the osseointegration, histological analysis was conducted. First, undecalcified sections were prepared to observe the formation of newborn bone surrounding the implants through sequential fluorescent labeling and methylene blue-acid fuchsin staining. Figures 8A and S10 reveal that there were more stained bones in the SPCP group, followed by the SPC group, suggesting that the two groups had strong osteogenic ability. Methylene blue-acid fuchsin staining was employed to label the newly formed bone integrated onto the implants. As presented in Fig. 8B, the new bone was tightly adhered to the porous surfaces of implants in the SPC and SPCP groups, indicating that they integrated well with the host bone and induced remarkable bone regeneration. In contrast, there was a large gap between the implant and the adjacent bone tissue in the PEEK group, reflecting the poor osteoinduction and osseointegration. Similarly, a gap was also observed in the SP group, although sulfonation treatment reduced the gap distance to a certain extent.

**Fig. 9:**
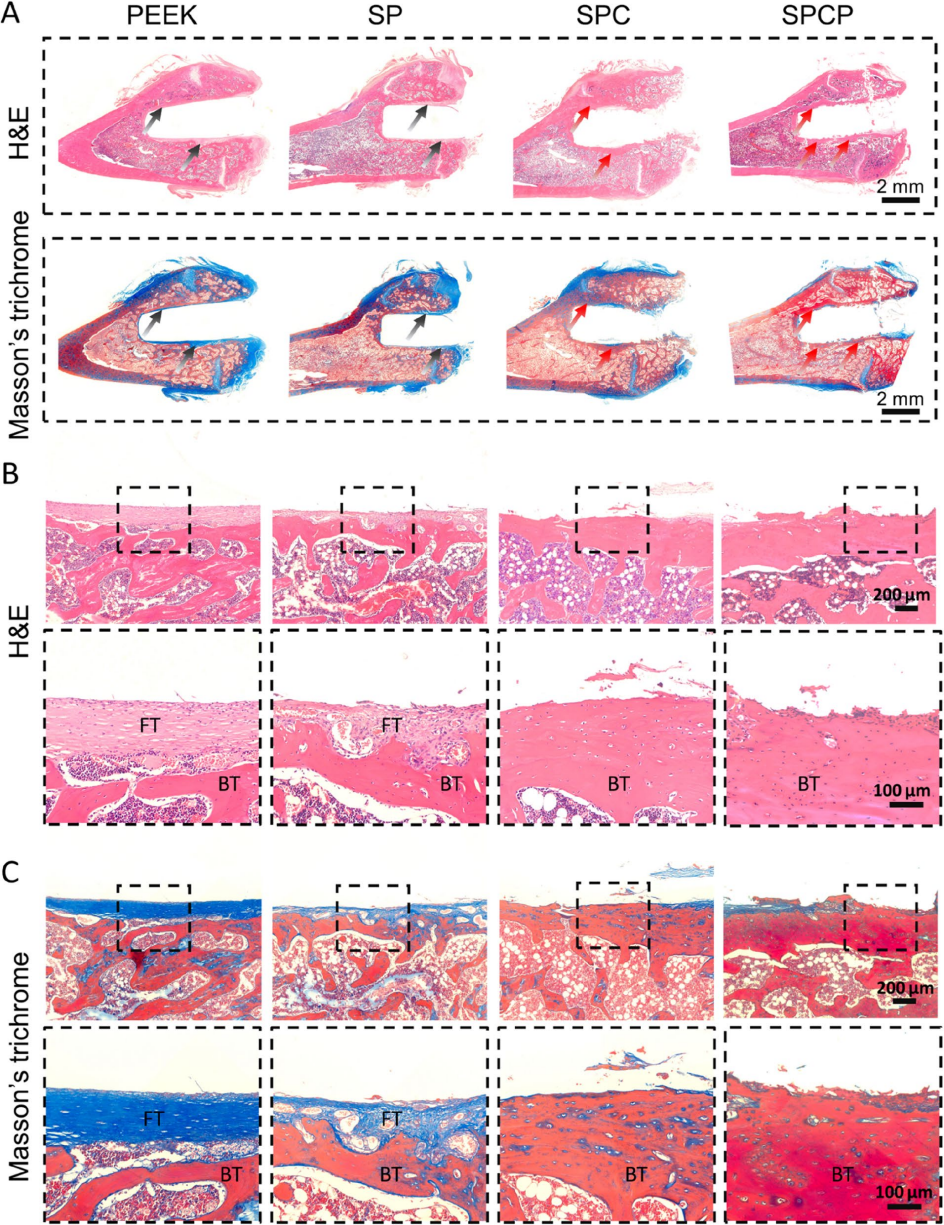
Histological evaluation of formation of newborn bone after various treatments. **A** Representative panoramic histological sections stained with H&E and Masson’s trichrome. The black arrows indicate FT, and the red arrows indicate newly formed bone. Local histological sections stained with **B**) H&E and **C**) Masson’s trichrome at different magnifications. FT: fibrous tissue; BT: bone tissue

Subsequently, decalcified sections were also obtained, and H&E and Masson’s trichrome staining were performed. H&E and Masson’s trichrome staining images showed that a layer of dense fibrous tissue (FT) enclosed the surface of PEEK implant (Fig. 9). This was attributed to the poor osteogenesis ability of the unmodified PEEK surface, which induced the FT ingrowth. Compared with the PEEK group, sulfonation treatment decreased the FT thickness adjacent to the SP implants and the FT formed was looser. Unlike the PEEK and SP groups, a large amount of dense, red-stained new bone tissue was seen at the periphery of the SPC and SPCP implants, which was congruent to the outcomes of μ-CT observation and toluidine blue-acid fuchsin staining.

**Fig. 10:**
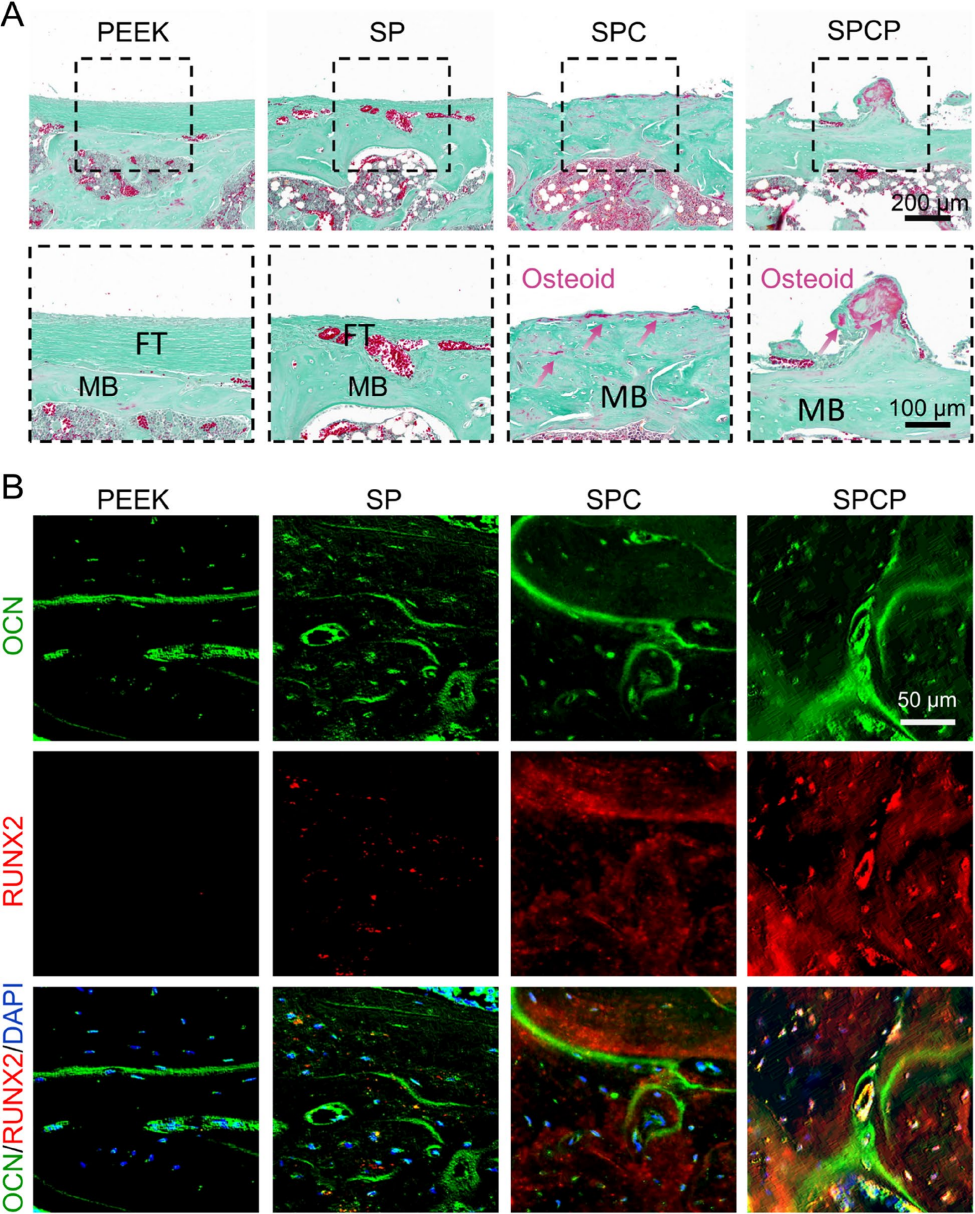
Histomorphological examination of the peri-implant region after various treatments. **A** Representative Goldner’s trichrome stained sections. The red arrows indicate unmineralized osteoid. MB: mineralized bone. **B** Representative immunofluorescence images of RUNX2 and OCN proteins

Furthermore, Goldner’s trichrome staining was also employed to detect the formation of new bone. As depicted in Fig. 10A, some unmineralized osteoid (orange/red staining) was observed around the SPC and SPCP implants. Conversely, almost no apparent immature bone was observed in the PEEK and SP groups.

Finally, we performed immunofluorescence staining of RUNX2 and OCN proteins to further verify bone regeneration in the peri-implant area at 8 weeks after implantation. RUNX2, an important transcription marker of osteogenesis, is generally expressed at the early stage of osteogenic differentiation, while OCN is a late osteogenic differentiation indicator [42]. As illustrated in Fig. 10B, the expression of OCN protein was high in all groups and the expression of RUNX2 protein was also remarkably upregulated in the SPC and SPCP groups. However, there was almost no apparent RUNX2-positive staining in the PEEK group and only weak RUNX2-positive staining in the SP group. This finding indicated that pristine PEEK surface had poor bone regeneration due to its biological inertia, and sulfonation treatment only improved the osteogenic activity of PEEK surface to some extent. In contrast, the SPC and SPCP implants exhibited high bone remodeling activity, which was coincident with histological observations. Taken together, these results revealed that the introduction of 3D hierarchical porous structure on the surface of PEEK-based implants could substantially promote new bone formation and osseointegration.

### Fabrication of surface modified PEEK-based intervertebral fusion device

**Fig. 11:**
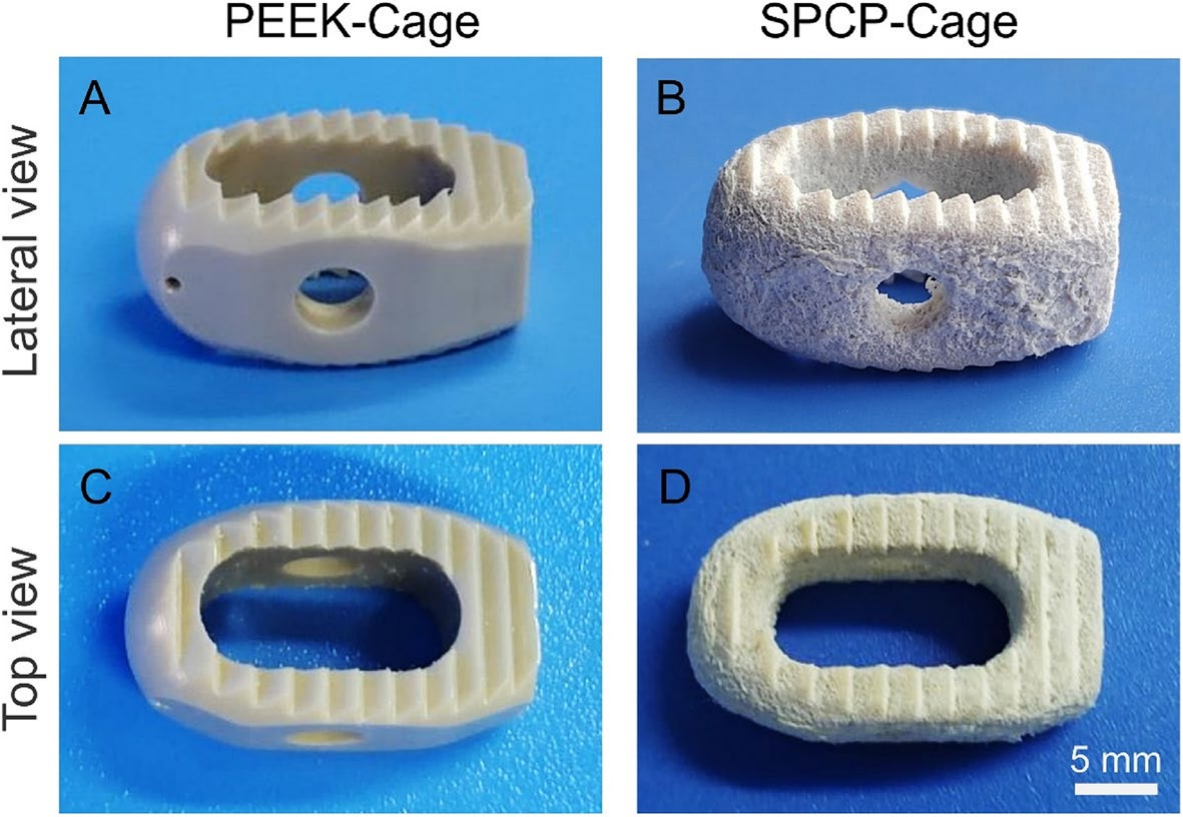
Surface modification of PEEK-based intervertebral fusion cage. **A**, **B** Lateral view and **C**, **D**) Top view of PEEK-cage and SPCP-cage. PEEK-cage: unmodified cage, SPCP-cage: cage surface with 3D hierarchical porous structure coupled with low-temperature oxygen plasma treatment

PEEK has been extensively used to the construction of a variety of orthopedic implants and in particular has the largest market share in interbody fusion cages [14]. To demonstrate the universality of the modification strategy developed by us, the surface of a medical intervertebral fusion device was also decorated. As presented in Figs. 11 and S11, compared with unmodified PEEK-cage with smooth surface, the surfaces of SPC-cage and SPCP-cage became rough obviously after sulfonation plus “cold pressing” treatment. The successful trial of this surface modification technology on intervertebral fusion cages showed its potential for future clinic applications.

## Discussion

Osseointegration refers to the ability of an implant to anchor with the surrounding new bone tissue without forming fibrous tissue, which is the most critical factor for the long-term stability of orthopedic implants in vivo [19, 32]. Due to the inherent bioinertness of PEEK, the fiber cysts generated after implantation hinder the direct contact between the implant and the surrounding bone, which inevitably leads to the implant loosening or even failure [18, 19]. Surface modification is an efficient means to improve the surface mechanical and biological performances of materials while maintaining their bulk properties [59–63]. Therefore, various surface modification ways, such as incorporation of bioactive coatings [25, 64], surface patterning [65, 66], surface roughness [22, 67], and construction of porous structure [32, 49], have been exploited to overcome the poor bone integration of conventional PEEK implants.

Considering that the natural bone tissue owns a hierarchical microporous structure with pore size of 10 to 400 μm [68], mimicking such an architecture has been regarded an appealing strategy to accelerate early implant integration with the surrounding bone [28, 69]. Inspired by this, a 3D hierarchical porous structure on PEEK surface was facilely created by sulfonation combined with “cold pressing” treatment without the need of special equipment in this study (Fig. 1A). The sulfonation treatment formed a small pore structure of 0.5–10 μm (Fig. 2A), which is conducive to cell adhesion [31], while the “cold pressing” treatment in the presence of NaCl porogenic agent plus subsequent particle leaching induced the formation of 100–200 μm large pore on the PEEK surface (Fig. 2A), which accommodates cell/tissue ingrowth. Moreover, the macropore structure on the PEEK surface could be easily tailored to meet different application scenarios by merely changing the size of porogenic agent (Fig. S4B).

In fact, surface roughness also plays an important role in promoting implant osseointegration [11, 22], and many studies have demonstrated that increased surface roughness can improve the osteogenic activity of PEEK implants [11, 22, 67, 70, 71]. Sandblasting and femtosecond laser treatment are commonly used methods to increase the surface roughness of implants [67, 70, 72]. However, the surface of implants treated by these methods usually only formed pits or small pores [67, 71]. Conversely, the sulfonation combined with “cold pressing” treatment not only substantially boosted the surface roughness of SPC and SPCP (Figs. 2B and 3A), but also formed a 3D hierarchical porous structure on their surfaces (Fig. 2A). This hierarchical topological structure provides more contact area with the host bone and facilitates tissue ingrowth, which is expected to form a stronger mechanical interlocking with the newly formed bone tissue.

Evans et al. also developed a surface-porous PEEK by hot pressing at 363 °C and 260 MPa in the presence of NaCl porogenic agent [30]. Nonetheless, due to the smooth surface of the NaCl particulate and its cubic feature, the PEEK surface lacked the small pore structure of 0.5–10 μm. In contrast, sulfonation treatment not only induced the small pore formation on the PEEK surface, but also softened the substrate, so that the NaCl porogenic agent could be conveniently embedded into the PEEK surface at ordinary temperature and pressure. Owing to the formation of small porous structure after sulfonation, sulfuric acid is easily retained, which has a negative impact on cells [34]. Therefore, hydrothermal treatment was employed to remove the residual sulfuric acid. As detailed in Fig. S5, the S content diminished sharply from 8.87 wt% before treatment to 1.12 wt% after treatment. Because sulfonation treatment can form -SO_3_H groups on the side chains of PEEK polymers [31], the remaining S is attributed to the formed -SO_3_H groups, which was also demonstrated via FTIR analysis (Fig. 3B). Meanwhile, the -SO_3_H groups on the PEEK surface are negatively charged, which can also improve the antibacterial ability of the substrate surface [34].

As expected, the surface modification did not significantly damage the mechanical performance of SPC and SPCP samples (Figs. 3E-F and S7). However, the sulfonation and “cold pressing” treatment resulted in an increase in the surface hydrophobicity of SPC, which was not conducive to the adhesion and spreading of BMSCs (Figs. 3C and 4A-B). Plasma treatment substantially boosted the surface hydrophilicity of SPCP, which promoted protein adsorption and facilitated cellular adhesion and spreading (Figs. 3C, 4A-B and 5B). Likewise, the adhesion state of BMSCs on the SP substrate was slightly better compared with the PEEK and SPC groups. This is due to the microporous structure formed by sulfonation [31]. Various surface modifications had no obvious effect on the proliferation of BMSCs compared with unmodified PEEK (Fig. 5A, C). Moreover intriguingly, the hierarchical porous structure of SPC and SPCP did improve cellular ingrowth (Figs. 5D and S8). In vitro osteogenic differentiation experiments further manifested that the ALP activity of BMSCs on SP, SPC and SPCP, especially SP and SPCP, was markedly higher than that of pristine PEEK after 7 days of incubation (Fig. 6A-B). However, the results of ARS staining showed that the in vitro mineralization effect of BMSCs on SPC and SPCP was significantly superior to PEEK and SP (Fig. 6A, C). This feature indicated that the early osteogenic activity was mainly dependent on the initial adhesion and viability of BMSCs, while the hierarchical porous structure, rather than surface hydrophilicity, played a crucial role in the maturation of mineralized nodules in the later stage.

Finally, in vivo experiments revealed that the presence of this hierarchical porous structure prominently improved the osteoinduction and osseointegration of PEEK implants. For example, μ-CT observation showed that both the SPC and SPCP implants induced more new bone formation and had higher bone-implant contact rate and their newborn bone had more Tb.N and smaller Tb.Sp compared with the pristine PEEK and SP implants (Fig. 7B-H). Histological analysis further substantiated that the newly formed bone in the SPC and SPCP groups was tightly attached to the implant surface, indicating the good bone integration (Fig. 8B). Conversely, due to the poor osteogenic activity of smooth PEEK surface, a dense fibrous layer formed at the periphery of implant in the PEEK group (Fig. 9), which inevitably affected bone regeneration and osseointegration. Sulfonation treatment could improve the effect of osseointegration to some extent, but it was still evidently inferior to the SPC and SPCP groups (Figs. 8, 9 and 10). Consequently, these results collectively confirmed that hierarchical topography played a central role in promoting implant bone integration.

## Conclusions

In this study, we proposed a simple but effective surface modification strategy, which combined sulfonation and “cold pressing” treatment to form a 3D hierarchical porous structure on the surface of PEEK implants. The technology was easy to operate, and the macropore size could be conveniently adjusted by changing the size of porogenic agent. The hierarchical topological structure on the SPC and SPCP surfaces not only facilitated the cellular ingrowth but also boosted the in vitro osteogenic differentiation and mineralization of BMSCs. The results of distal femoral defect repair in SD rats showed that the SPC and SPCP implants substantially promoted new bone formation and accelerated osseointegration. Overall, the present study indicates that the hierarchical porous surface topology can greatly improve implant-bone integration. In addition, the successful demonstration of this surface modification technique on a medical PEEK interbody fusion cage further shows its practical application potential.

## Supplementary Information

**Additional file 1: Fig. S1.** Schematic illustration of preparing PEEK samples by hot pressing molding.** Fig. S2.** Schematic illustration of extracting BMSCs from SD rat femur.** Table S1.** Primer sequences of qRT-PCR analysis for the mRNA expression.** Fig. S3.** SEM images of PEEK surfaces after different times of sulfonation combined with “cold pressing” treatment.** Fig. S4.** SEM images of modified surfaces. A) Cross sections of the indicated samples; B) Macropore size adjusted by different sizes of porogenic agents.** Fig. S5.** EDS spectra for S element of A) PEEK, B) SP’ and C) SP. SP’ means the sample before hydrothermal treatment. D) Semi-quantitative results of S content.** Fig. S6.** Representative optical images of affinity of different surfaces to water and diiodomethane.** Fig. S7.** Shore hardness of different samples.** Fig. S8.** Z-directional CLSM images of BMSCs on various samples at 4 and 7 days after culturing.** Fig. S9.** Quantitative analysis of μ-CT data including A) BS/TV and B) Tb.Th.** Fig. S10.** CLSM images of different fluorescent dye labels around implants. The dashed lines show the boundary between the implant and the surrounding tissue.** Fig. S11.** Global views of the cages with modified surfaces. SP-cage: sulfonation treatment, SPC-cage: surface with 3D hierarchical porous structure.

## Data Availability

The datasets used and analyzed during the current study are available from the corresponding author on reasonable request.

## Abbreviations

PEEK: Polyetheretherketone
BMSCs: Bone marrow-derived mesenchymal stem cells
NaCl: Sodium chloride
SD: Sprague Dawley
SP: Sulfonation and hydrothermal treatment
SPC: Cold pressing and hydrothermal treatment of SP
SPCP: Low-temperature oxygen plasma treatment of SPC
2D: Two-dimensional
3D: Three-dimensional
FTIR: Fourier transform infrared spectroscopy
FE-SEM: Field emission scanning electron microscope
FN: Fibronectin
PBS: Phosphate buffered saline
SDS: Sodium dodecyl sulfate
FBS: Fetal bovine serum
α-MEM: Minimum essential medium α
DAPI: (2-(4-Amidinophenyl)-6-indolecarbamidine dihydrochloride
CLSM: Confocal laser scanning microscope
PI: Propidium iodide
CCK-8: Cell counting kit-8
ALP: Alkaline phosphatase
pNPP: Para-nitrophenyl phosphate
ARS: Alizarin red-S
qRT-PCR: Quantitative reverse transcription polymerase chain reaction
OPN: Osteopontin
OCN: Osteocalcin
BSP: Bone sialoprotein
ROI: Region of interest
BIC: Bone-implant contact
TV: Total volume
BV: Bone volume
BS: Bone surface
BMD: Bone mineral density
Tb.N: Trabecular bone number
Tb.Sp: Trabecular separation
Tb.Th: Trabecular thickness
EDTA: Ethylenediaminetetraacetic acid
