# Supplementary material for: Polyetheretherketone implants with hierarchical porous structure for boosted osseointegration
Source: Biomater Res. 2023 Jun 27;27:61. doi: 10.1186/s40824-023-00407-5 (PMC10294516; doi:10.1186/s40824-023-00407-5)
Supplement: Supplementary file 1 — Additional file 1: Fig. S1. Schematic illustration of preparing PEEK samples by hot pressing molding. Fig. S2. Schematic illustration of extracting BMSCs from SD rat femur. Table S1. Primer sequences of qRT-PCR analysis for the mRNA expression. Fig. S3. SEM images of PEEK surfaces after different times of sulfonation combined with “cold pressing” treatment. Fig. S4. SEM images of modified surfaces. A) Cross sections of the indicated samples; B) Macropore size adjusted by different sizes of porogenic agents. Fig. S5. EDS spectra for S element of A) PEEK, B) SP’ and C) SP. SP’ means the sample before hydrothermal treatment. D) Semi-quantitative results of S content. Fig. S6. Representative optical images of affinity of different surfaces to water and diiodomethane. Fig. S7. Shore hardness of different samples. Fig. S8. Z-directional CLSM images of BMSCs on various samples at 4 and 7 days after culturing. Fig. S9. Quantitative analysis of μ-CT data including A) BS/TV and B) Tb.Th. Fig. S10. CLSM images of different fluorescent dye labels around implants. The dashed lines show the boundary between the implant and the surrounding tissue. Fig. S11. Global views of the cages with modified surfaces. SP-cage: sulfonation treatment, SPC-cage: surface with 3D hierarchical porous structure. [file 40824_2023_407_MOESM1_ESM.docx]

# Supporting information

# Polyetheretherketone implants with hierarchical porous structure for boosted osseointegration

Zhiyong Chen^1^, Yu Chen^1^, Yang Wang^1^, JiaJia Deng^2^, Xin Wang^1^, Qingqing Wang^3^, Yuehua Liu^2^, Jiandong Ding^1^ and Lin Yu^1,^*

^1^State Key Laboratory of Molecular Engineering of Polymers, Department of Macromolecular Science, Shanghai Stomatological Hospital & School of Stomatology, Fudan University, Shanghai 200438, China

^2^Department of Orthodontics, Shanghai Stomatological Hospital & School of Stomatology, Shanghai Key Laboratory of Craniomaxillofacial Development and Diseases, Fudan University, Shanghai 200001, China

^3^Department of Orthopaedic Surgery, Sir Run Run Shaw Hospital, Medical College of Zhejiang University, Hangzhou, Zhejiang 310016, China.

* Corresponding author

E-mail address: yu_lin@fudan.edu.cn

This file includes Figs. S1-S11 and Table S1. The supplementary figures and tables are shown in the sequence mentioned in the main manuscript.


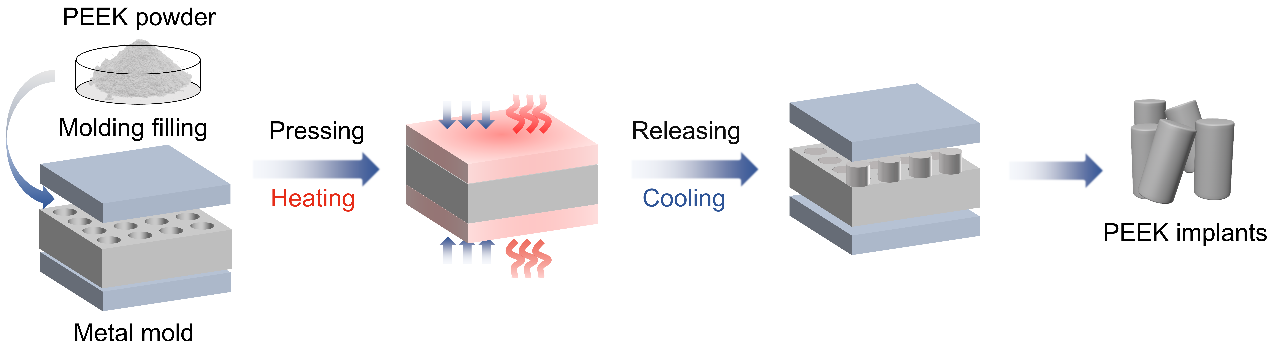


**Fig. S1** Schematic illustration of preparing PEEK samples by hot pressing molding.


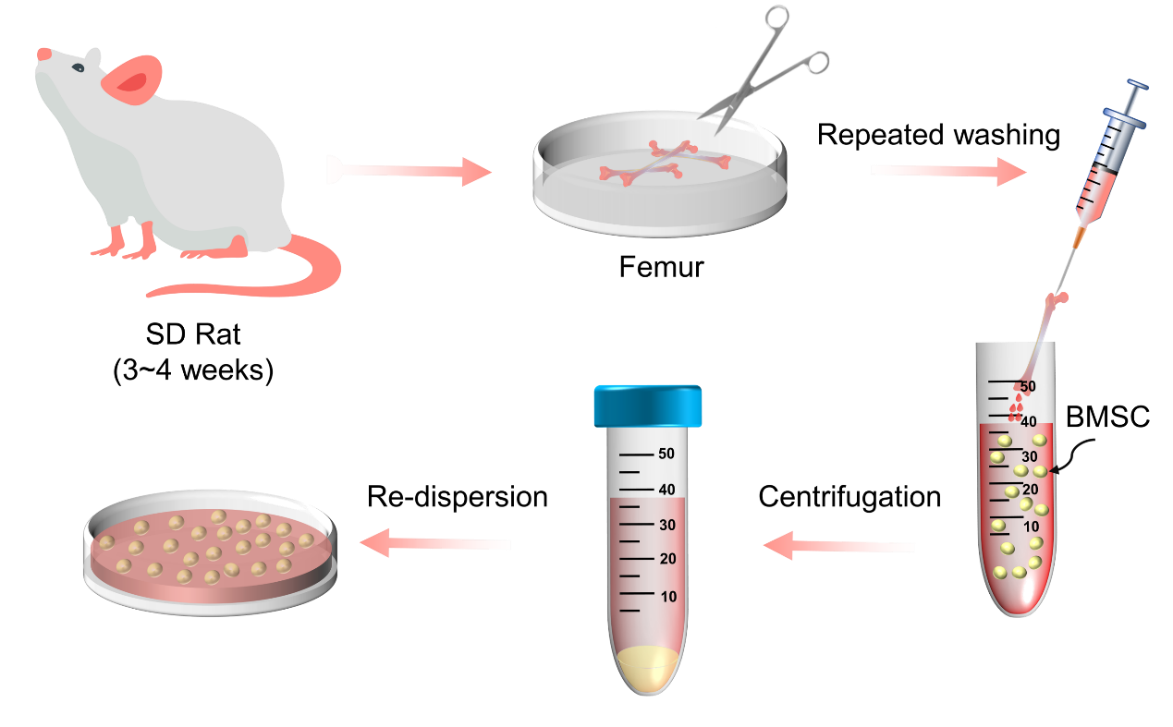


**Fig. S2** Schematic illustration of extracting BMSCs from SD rat femur.

**Table S1.** Primer sequences of qRT-PCR analysis for the mRNA expression.

| Genes | Primers (5′-3′) | |
| --- | --- | --- |
| OPN | F: CCAGCCAAGGACCAACTACA | R: AGTGTTTGCTGTAATGCGCC |
| OCN | F: GCAGACCTAGCAGACACCAT | R: TTGGACATGAAGGCTTTGTCA |
| BSP | F: CCAGCCAAGGACCAACTACA | R: AGTGTTTGCTGTAATGCGCC |
| β-actin | F: CCCGCGAGTACAACCTTCTTG | R: GTCATCCATGGCGAACTGGTG |


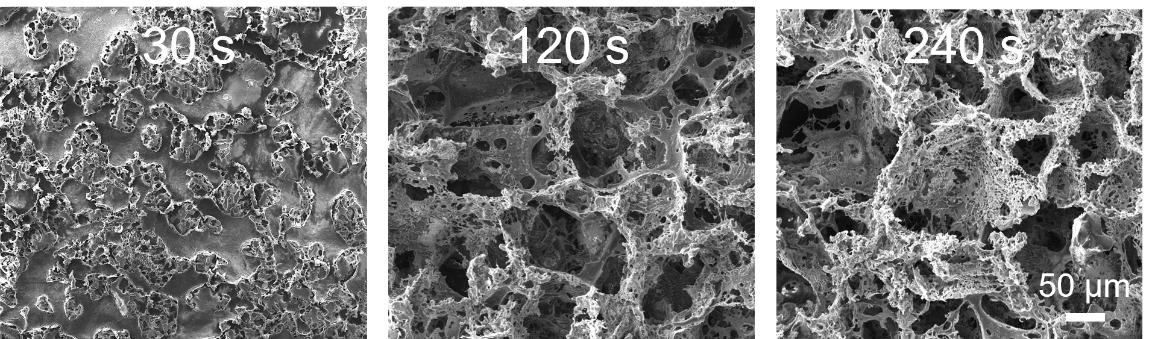


**Fig. S3** SEM images of PEEK surfaces after different times of sulfonation combined with “cold pressing” treatment.


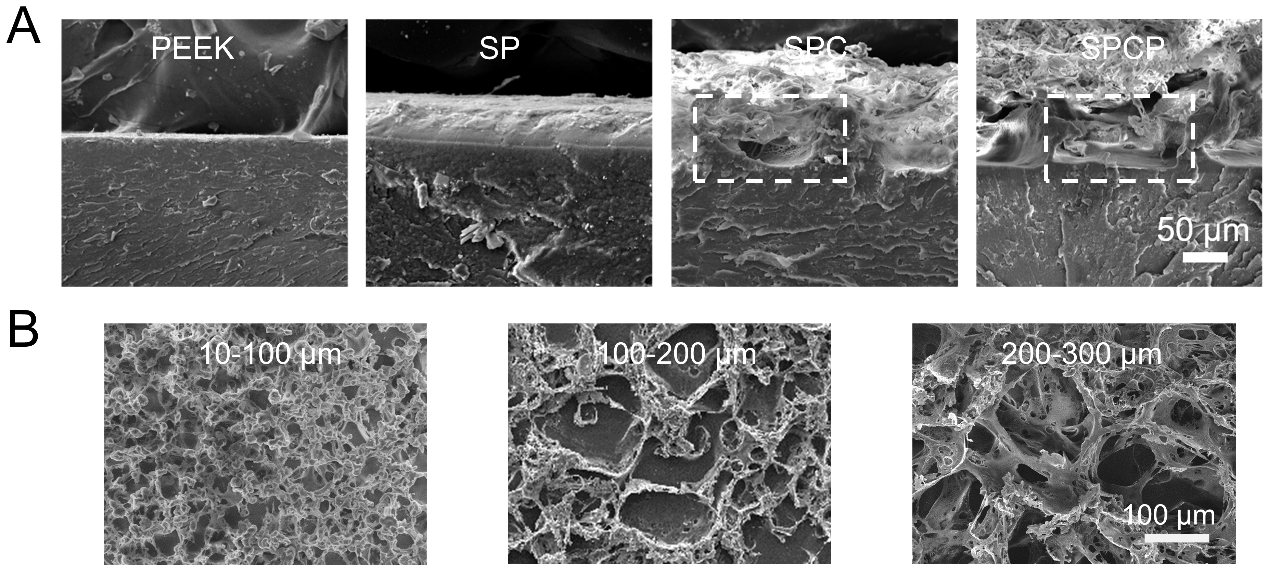


**Fig. S4** SEM images of modified surfaces. A) Cross sections of the indicated samples; B) Macropore size adjusted by different sizes of porogenic agents.


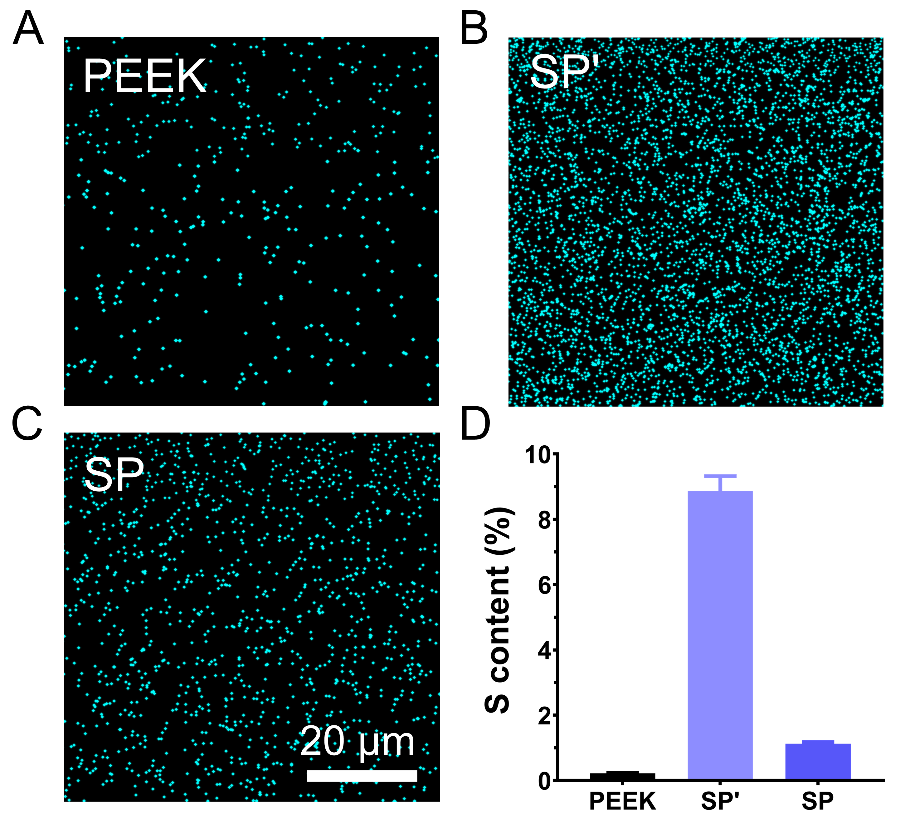


**Fig. S5** EDS spectra for S element of A) PEEK, B) SP’ and C) SP. SP’ means the sample before hydrothermal treatment. D) Semi-quantitative results of S content (*n* = 3).


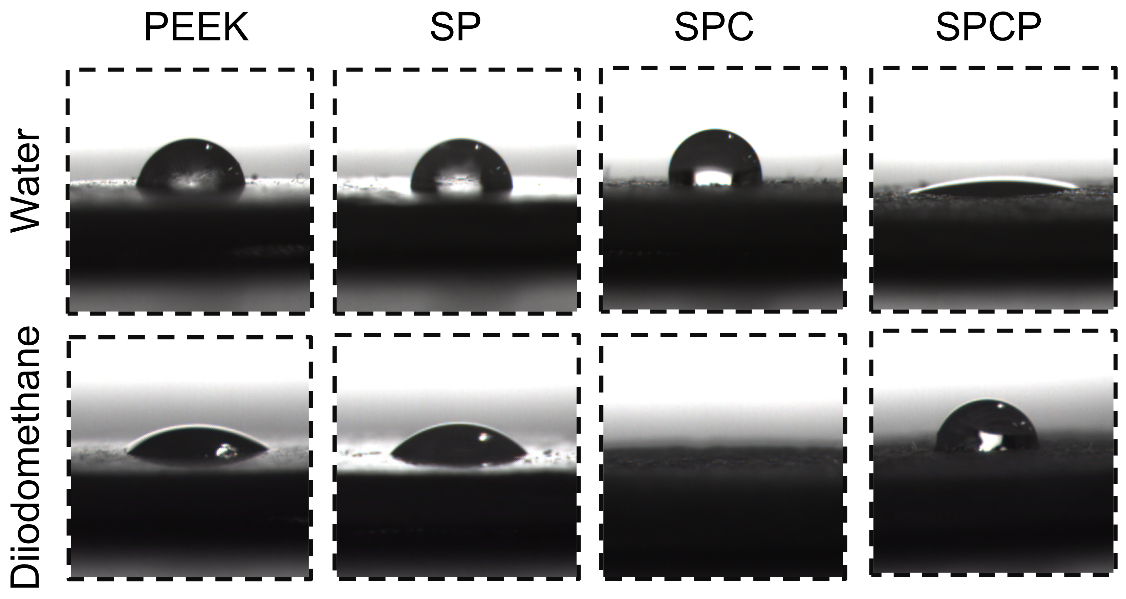


**Fig. S6** Representative optical images of affinity of different surfaces to water and diiodomethane.


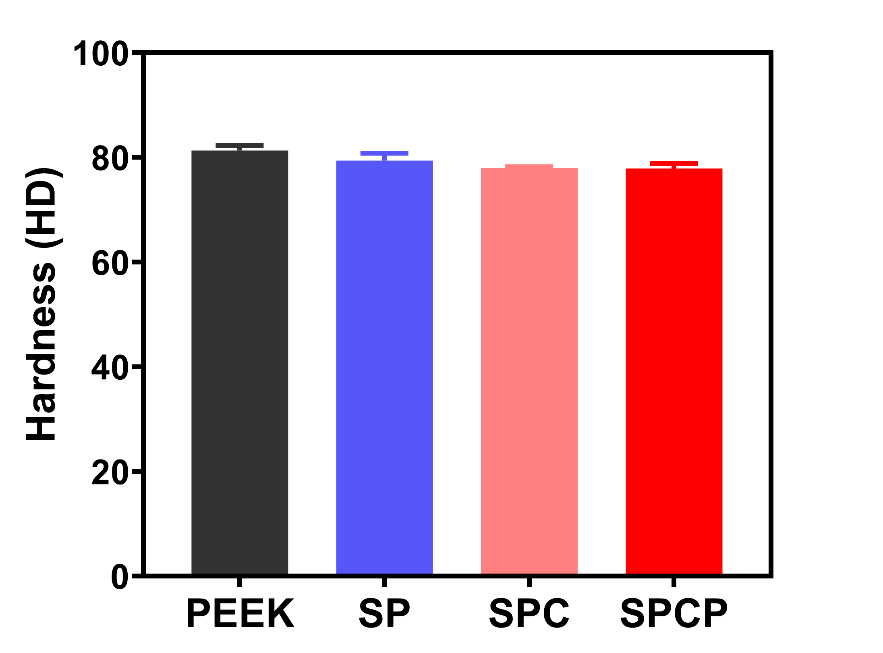


**Fig. S7.** Shore hardness of different samples (n = 4).


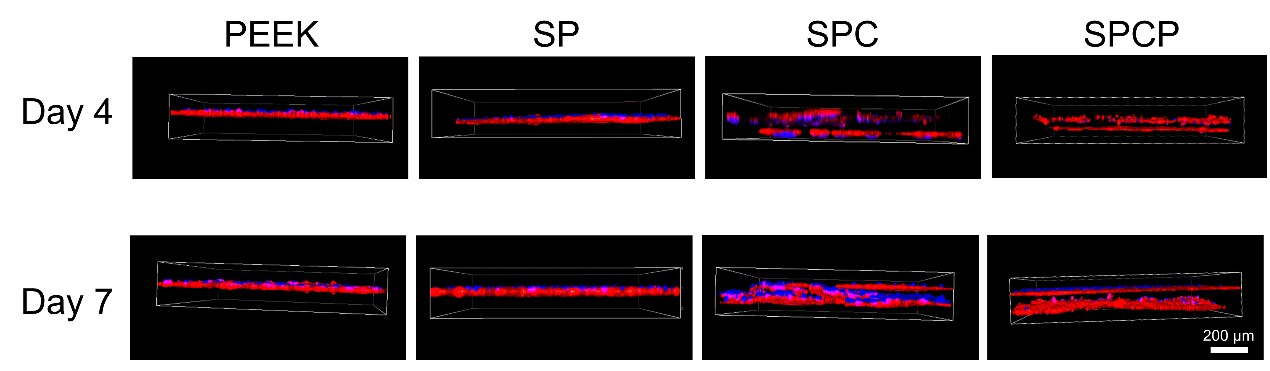


**Fig. S8** Z-directional CLSM images of BMSCs on various samples at 4 and 7 days after culturing. (Blue: cell nuclei stained by DAPI, Red: cytoskeleton stained by rhodamine-phalloidin)


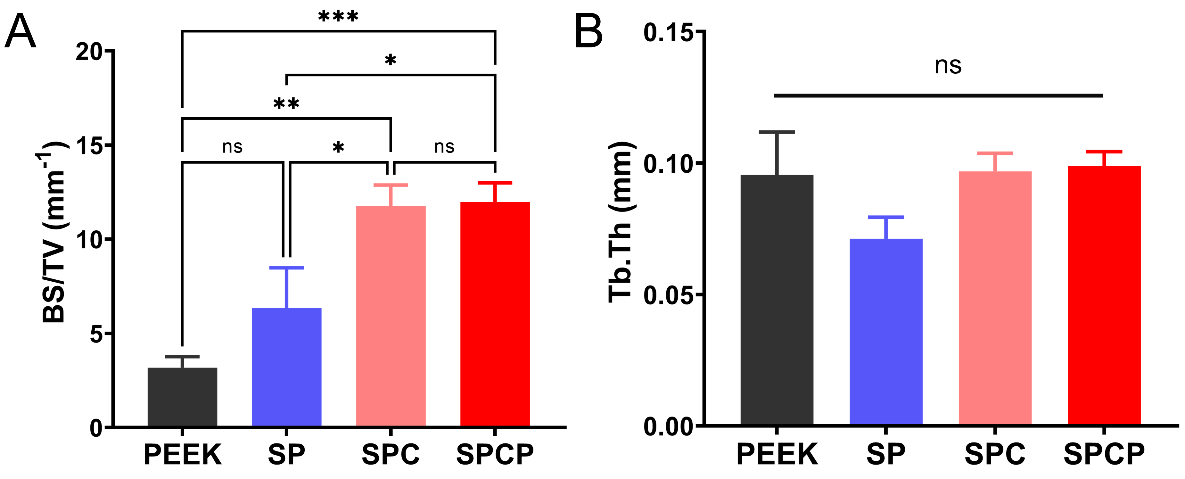


**Fig. S9** Quantitative analysis of μ-CT data including A) BS/TV and B) Tb.Th (n = 6).


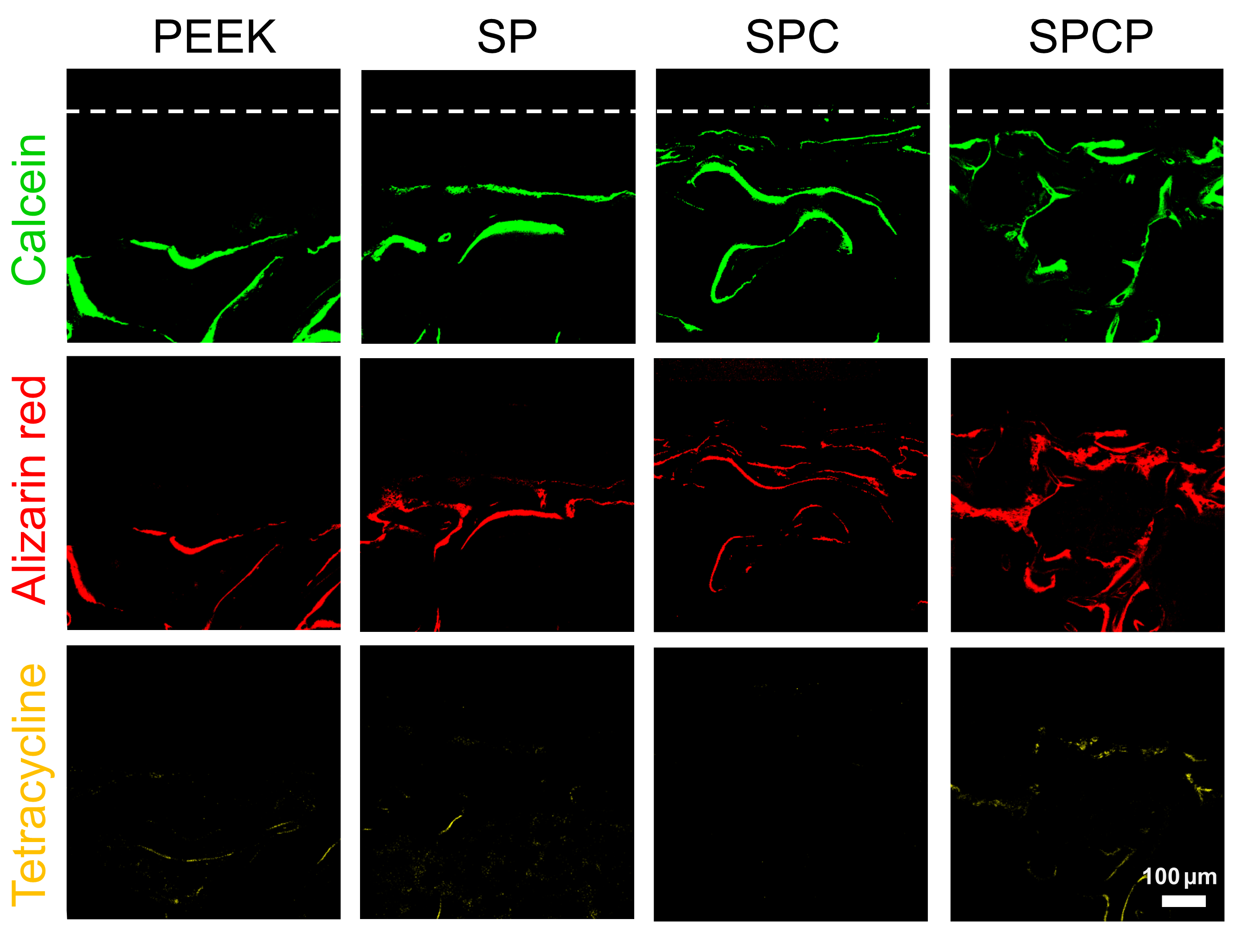


**Fig. S10** CLSM images of different fluorescent dye labels (Green: Calcein; Red: Alizarin red; Yellow: Tetracycline) around implants. The dashed lines show the boundary between the implant and the surrounding tissue.


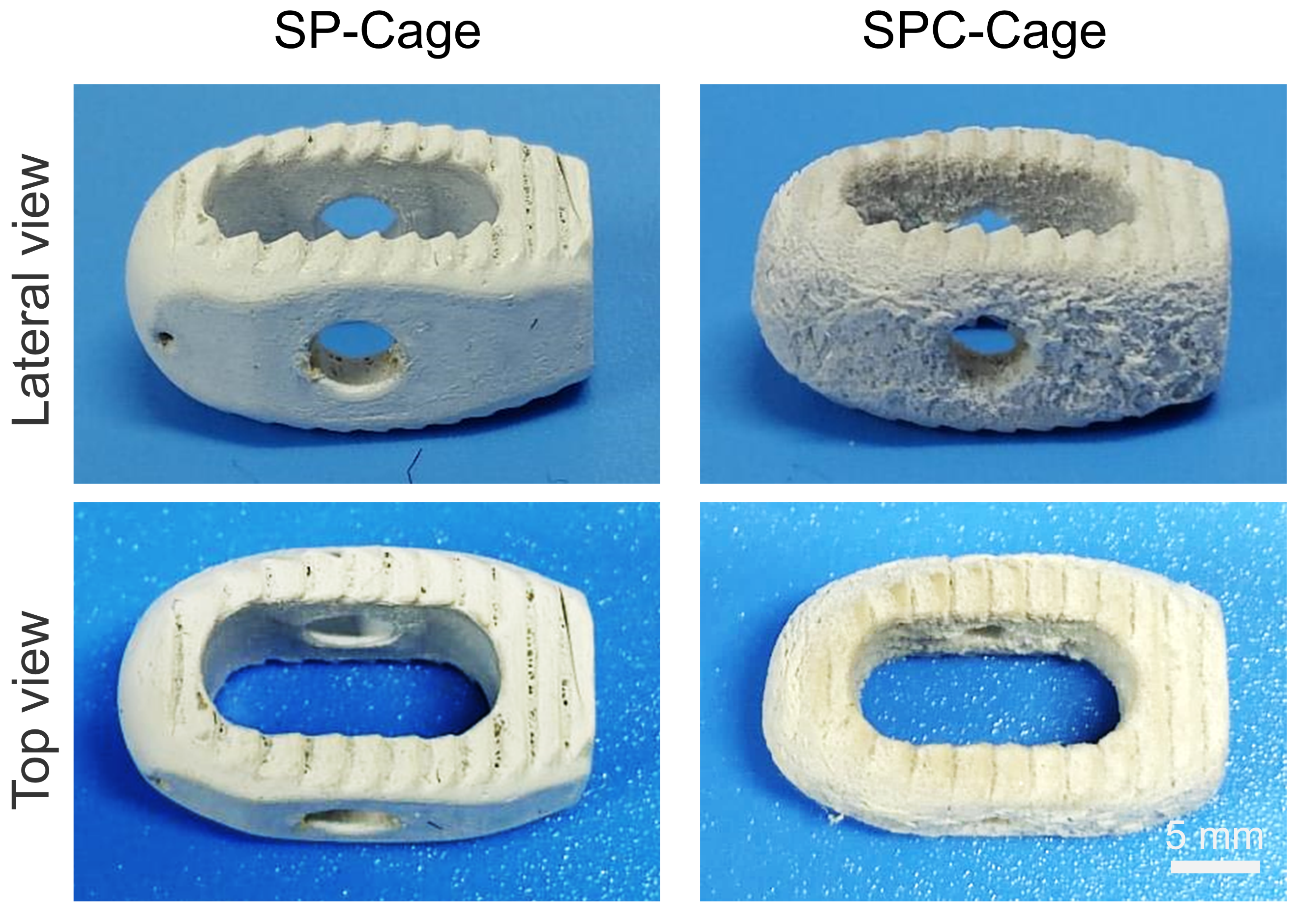


**Fig. S11** Global views of the cages with modified surfaces. SP-cage: sulfonation treatment, SPC-cage: surface with 3D hierarchical porous structure.
